# Exploring the Prototypical Definitions of Intelligent Engineers Held by Irish and Swedish Higher Education Engineering Students

**DOI:** 10.1177/00332941211000667

**Published:** 2021-03-12

**Authors:** Jeffrey Buckley, Tomás Hyland, Lena Gumaelius, Niall Seery, Arnold Pears

**Affiliations:** Department of Learning, KTH Royal Institute of Technology, Sweden; Faculty of Engineering and Informatics, 8823 Athlone Institute of Technology, Ireland; Faculty of Engineering and Informatics, 8823 Athlone Institute of Technology, Ireland; Department of Learning, KTH 7655 Royal Institute of Technology, Sweden; Office of the President, 8823 Athlone Institute of Technology, Ireland; Department of Learning, KTH 7655 Royal Institute of Technology, Sweden

**Keywords:** Higher education, diversity, engineering education, intelligence, field-specific ability beliefs, culture

## Abstract

Males are generally overrepresented in higher education engineering. However, the magnitude of this variance differs between countries and engineering fields. Evidence associated with the field-specific ability beliefs hypothesis suggests that perceptions of intelligence held by actors within engineering affects the engagement of underrepresented groups. This study examined perceptions of an intelligent engineer held by undergraduate and postgraduate engineering students in Ireland and Sweden, countries selected based on their levels of female representation in engineering education. It was hypothesised that there would be a significant difference in perceptions between countries. A survey methodology was employed in which a random sample of Irish and Swedish university students completed two surveys. The first asked respondents to list characteristics of an intelligent engineer, and the second asked for ratings of importance for each unique characteristic. The results indicate that an intelligent engineer was perceived to be described by seven factors; practical problem solving, conscientiousness, drive, discipline knowledge, reasoning, negative attributes, and inquisitiveness when the data was analysed collectively, but only the five factors of practical problem solving, conscientiousness, drive, discipline knowledge and negative attributes were theoretically interpretable when the data from each country was analysed independently. A gender × country interaction effect was observed for each of these five factors. The results suggest that the factors which denote intelligence in engineering between Irish and Swedish males and females are similar, but differences exist in terms of how important these factors are in terms group level definitions. Future work should consider the self-concepts held by underrepresented groups with respect to engineering relative to the factors observed in this study.

## Introduction

Higher education engineering fields are generally male dominated (Cheryan et al., 2011, 2017; Hunt, 2016; Sultan et al., 2018; Sunny et al., 2017; Verdín et al., 2018; Wiebe et al., 2018; Yoder, 2017). This is problematic as a lack of diversity suggests a loss of potential talent, and the gender disparity indicates the existence of entry barriers to women. Attracting and promoting diverse talent is a critical agenda of engineering fields to ensure their growth and prosperity and it is paramount that all individuals have the opportunity to form and pursue their own aspirations without negative impacts from prejudice or bias. In order to fully understand the issue of gender disparity and to progress it, it is imperative that the nuances associated with gender representation in higher education engineering are understood, and particularly if there are differences between countries that could impact the generalisability of research findings.

Wang and Degol (2017) summarised six explanations provided within the pertinent literature for the existence of gender representation gaps in science, technology, engineering and mathematics (STEM) fields. One of these, the field-specific ability beliefs hypothesis, suggests that women may be underrepresented in academic disciplines where success is believed to be more reliant on innate brilliance as opposed to an investment of effort (Leslie et al., 2015, p. 262). Considering that women tend to judge themselves more harshly than men (Blatchford, 1997; Langan et al., 2008; Torres-Guijarro & Bengoechea, 2017), and the calls to identify relevant elements of self-concept (Sax, 1994; Sax et al., 2015), identifying field-specific characteristics perceived to be associated with success and exploring self-perceptions with respect to these characteristics may provide further insight into ways to address the gender gap in higher education engineering. Specifically, having an understanding of such field-specific characteristics would allow for investigations into people’s self-rating of those characteristics and how, in relation to their gender and culture, these relate to desirable outcomes such as interest, motivation and performance.

### Current study

Gender in engineering education has been explored through a number of lenses, however there is a need for greater diversity in this regard (Pawley et al., 2016). In response to the gender gap in third level engineering and stemming from the field-specific ability beliefs hypothesis, this study aimed to identify and determine the importance of perceived characteristics of an intelligent engineer from the perspective of higher education engineering students across two countries, Ireland and Sweden, using a survey methodology pioneered by Sternberg et al. (1981). Building on the work of Adams et al. (2007) who explored Swedish students conceptions of engineering as a discipline, having an understanding of the characteristics perceived to be associated with intelligent engineers would facilitate more explicit investigations into whether young people’s self-concepts of these characteristics are related to their engagement with engineering. Irish and Swedish students were selected primarily based on the variance in female representation in engineering education between them. Data from the Organisation for Economic Co-operation and Development (OECD, 2020) indicates that the percentage of females enrolled in “engineering and engineering trades” education at bachelor’s, master’s and doctoral level ranges from 11.54% to 28.33% in OECD countries, and that Sweden has the highest percent of female representation (28.33%) whilst Ireland has one of the lowest (14.33%). Third level engineering students were included as participants as they were actively in the environment that the gender gap relates to and thus offered a critical perspective. In investigating the prototypical definitions (Neisser, 1979; Rosch, 1977; Rosch et al., 1976; Rosch & Mervis, 1975) of intelligent engineers held by the participating students, the study had the following objectives:

- To elicit Irish and Swedish engineering students’ perceptions of the characteristics that describe an intelligent engineer.
- Based on these characteristics, to identify broad factors through an exploratory factor analysis (EFA) perceived to reflect an intelligent engineer.
- To examine differences in rated importance between these factors relative to the students’ conceptions of an intelligent engineer.
- To further examine the differences in the rated importance relative to the students self-reported gender and country of study.
- As the study relates to addressing the gender gap in engineering education engagement, to examine of the differences in the rated importance of these factors between female Irish and Swedish students.

In presenting this study, first a theoretical background will be provided to give context on gender and culture in STEM and engineering, the field specific ability beliefs hypothesis, and prototypical definitions of intelligence. This will be followed by a description of the study method which involved the administration of two surveys which are presented consecutively. Finally, the results of this study will be discussed in light of cultural differences between Ireland and Sweden and with respect to relevant theory.

## Theoretical background

### Gender and culture in STEM and engineering

Eisenhart (2001) noted that if culture is related to a study’s design, it is important to define how culture is conceptualized within the study. Schein (1992, p. 1) defined culture for a group as:

… a pattern of shared basic assumptions that the group learned as it solved its problems of external adaptation and internal integration, that has worked well enough to be considered valid, and therefore, to be taught to new members as the correct way to perceive, think, and feel in relation to those problems.

In terms of engineering in higher education, Godfrey and Parker (2010) offer an overview of perspectives taken on studying culture which includes culture as gendered (Cronin & Roger, 1999), culture as an agent in student attrition (Courter et al., 1998), student engagement and enculturation (Volkwein et al., 2004), the development of engineering identity (Stevens et al., 2008), faculty cultures (McKenna et al., 2008), campus cultures (Tonso, 2006), sub-disciplinary cultures (Murphy et al., 2007), national cultures (Downey & Lucena, 2005), assessment cultures (Borrego, 2008), the role of institutional culture in effecting change (Covington & Froyd, 2004), and measuring cultural change (Fromm & McGourty, 2001). However, Godfrey and Parker (2010) also note that each of these perspectives only offer a partial view of the dimensions of culture in engineering education. In this study, as the countries of Ireland and Sweden are being compared, culture is conceptualised in terms of national cultures, and similar to Godfrey and Parker (2010, p. 7) the position taken in this study is that “culture is not static but open to shifting values and cultural norms. Any snapshot of a culture will therefore be situated at a particular place and time”.

There is an inherent limitation in considering culture at a national level, which is that countries have varying demographics of people, each with potentially unique sub-cultures. However, there are also advantages to considering culture at a national level in that results can provide empirical support for in-depth explorations into the impact of culture related variables with respect to within country demographics. Of the studies which consider culture at a national level in engineering education, relatively few are associated with comparisons between a small number of countries. Most pertinent studies consider an extensive range of countries or conduct an in-depth investigation into the culture of one country. Of those that consider culture nationally across a large range of countries, results of international assessments such as the Trends in International Mathematics and Science Study (TIMMS) and studies from the Programme for International Student Assessment (PISA) are often used (e.g., Charles et al., 2014; Hamamura, 2012; Mann & DiPrete, 2016; Nosek et al., 2009; Reilly, 2012; Stoet et al., 2016).

Many interesting findings have come from studies which have considered results from TIMMS and PISA with respect to variables associated with STEM and engineering interest and performance, such as that “contra predictions, economically developed and more gender equal countries have a lower overall level of mathematics anxiety, and yet a larger national sex difference in mathematics anxiety relative to less developed countries” and that “although relatively more mothers work in STEM fields in more developed countries, these parents valued, on average, mathematical competence more in their sons than their daughters. The proportion of mothers working in STEM was unrelated to sex differences in mathematics anxiety or performance” (Stoet et al., 2016, p. 1). Similarly, Mann and DiPrete (2016, p. 568) “demonstrate that girls hold themselves to a higher performance standard than do boys before forming STEM orientations, and this gender “standards gap” grows with the strength of a country’s performance environment [and] that a repeatedly observed paradox in this literature – namely, that the STEM gender gap increases with a more strongly gender-egalitarian national culture – vanishes when the national performance culture is taken into account”. Furthermore, Nosek et al. (2009) found that nation-level implicit stereotypes predicted nation-level sex differences in mathematics and science performance in 8th grade students. National differences in cognitive sex differences, another variable linked with STEM and engineering interest and performance (Wang & Degol, 2017) are also often cited within this literature. For example, Reilly (2012) illustrates that from the 2009 PISA results, across 34 countries, females generally outperformed males in reading, males generally outperformed females in mathematics, and there was a variance observed across countries for science performance. Results from these studies can have substantial benefit, as when results indicate commonality between countries, inferences can be made relating to variables which generalise across nations. However, when there are reported differences between countries, country cultural variables can be inferred to have causal implications and thus merit further confirmatory inquiry.

### The field-specific ability beliefs hypothesis

Much evidence indicates cultural associations between men and innate intelligence but not women (Bian et al., 2018; Elmore & Luna-Lucero, 2017; Kirkcaldy et al., 2007; Tiedemann, 2000), and women tend to be underrepresented in fields which are considered to require innate brilliance in comparison to those where the attainment of excellence or expertise is associated with effort. These stereotypes of women, in addition to work examining the variability in individuals beliefs about success (Dweck, 1999, 2006), underpinned the postulation of the field-specific ability beliefs hypothesis (Leslie et al., 2015). This is not to suggest that natural ability is or is not important to certain fields in reality, but rather this hypothesis is specifically associated with practitioners’ opinions concerning the importance of innate ability in the field they are working in. In a large scale study (n = 1820), Leslie et al. (2015) tested the field-specific ability beliefs hypothesis against three competing hypotheses; (1) the more demanding a discipline in terms of work hours, the fewer the women, (2) the more selective a discipline, the fewer the women, and (3) the more a discipline prioritizes systemizing over empathizing, the fewer the women. The results of their study supported the field-specific ability hypothesis over the other three, and that the hypothesis extended to the underrepresentation of African Americans’ as well. Critically, while the related and underpinning work associated with growth mindset theory has recently seen an emergence of contradictory evidence (Bahník & Vranka, 2017; Foliano et al., 2019; Li & Bates, 2017; Sisk et al., 2018), it is theoretically different than the field-specific ability beliefs hypothesis (e.g., Gunderson et al., 2017) which has corroborating evidence (Bian et al., 2018; Cimpian & Leslie, 2015; Deiglmayr et al., 2019; Storage et al., 2016).

There are several causal explanations associated with this hypothesis. In understanding these, the differences between overt/intentional and covert/subtle forms of sexism, and between hostile and benevolent forms of sexism must be considered (Swim et al., 1995, 2005; Swim & Cohen, 1997). Wang and Degol (2017) note that although overt and deliberate forms of discrimination may not be as common now as they used to be, covert and benevolent forms still exist and shape male and female career trajectories. Notably, research shows that children as young as 6 are influenced by gender stereotypes, such as that science and mathematics are male domains (Miller et al., 2015) and that boys are more likely to be “really, really smart” (Bian et al., 2017). One example of a causal explanation is related to perceived sense of community within fields. For example, Cheryan and Plaut (2010) found when studying English, a female-dominated field, and computer science, a male-dominated field, that “the best mediator of women’s lower interest in computer science and men’s lower interest in English was perceived similarity” (p.475). Furthermore, Cheryan et al. found that the removal of stereotypical masculine objects (e.g., Star Trek posters and video games) could increase female interest in these courses (Cheryan et al., 2009, 2011). Leslie et al. (2015) summarise additional causal mechanisms for the field-specific ability beliefs hypothesis, stating that:

The practitioners of disciplines that emphasize raw aptitude may doubt that women possess this sort of aptitude and may therefore exhibit biases against them (Valian, 1998). The emphasis on raw aptitude may activate the negative stereotypes in women’s own minds, making them vulnerable to stereotype threat (Dar-Nimrod & Heine, 2006). If women internalize the stereotypes, they may also decide that these fields are not for them (Wigfield & Eccles, 2000).

It should be noted that recent studies have failed to replicate stereotype threat effects (Finnigan & Corker, 2016; Flore et al., 2018; Sunny et al., 2017) however this was only one of the theorized causal relationships between the field-specific ability beliefs hypothesis and the gender disparities which exists in STEM areas. Considering the abundance of evidence illustrating either the lower self-concept reported by women, or the general under valuing of their self-reports (e.g., Blatchford, 1997; Langan et al., 2008; Torres-Guijarro & Bengoechea, 2017), there is still substantial merit in exploring young people’s self-perceptions relative to field-specific abilities in an attempt to understand gender representation in higher education. A final causal relationship stems from the empirical work of Bian et al. (2018). As a result of six studies they suggest that “portraying a profession as requiring brilliance undermines women's interest in [them]” (p. 419) positing the underpinning psychological processes to be related to their finding that “women were less sure of success in brilliance-oriented settings and believed they were dissimilar to the type of person who commonly works in these settings” (p. 418).

### Prototypical definitions of intelligence

There are multiple perspectives from which to view implicit theories of intelligence. The work associated with mindsets relates to beliefs about the nature of intelligence, i.e., if it is fixed or malleable. Relatedly, but theoretically different, are implicit theories regarding the structure of intelligence, i.e. what it means to be intelligent. Implicit theories of the structure of intelligence are important as explicit definitions of intelligence are widely varied, and “verbal definitions of the intelligence concept have never been adequate or commanded consensus” (Meehl, 2006, p. 435), and as such beliefs have real life implications, such as relating to the lower self-estimates made by women than men (Pérez et al., 2010). Based on the importance of understanding people’s implicit definitions of intelligence, Neisser (1979) describes the utility in considering prototypical definitions of the construct. Based on the work of Rosch with regards to categorization (Rosch, 1977; Rosch et al., 1976; Rosch & Mervis, 1975), Neisser (1979, p. 182) describes the prototype of a category or concept as being “that instance (if there is one) which displays all the typical properties”. In other words, generating a prototypical definition allows for a description to be established which puts forward its typical properties as decided upon by a specific cohort of people. So while a prototypical definition may not be an objectively valid definition of a construct, it has importance as it reflects the collective opinion of a specific group of people. A survey methodology to elicit prototypical definitions of intelligence was pioneered by Sternberg et al. (1981) whereby experts and laypeople were initially asked to list characteristics of intelligence, academic intelligence, everyday intelligence, and unintelligence. Following this, different samples from within the same demographic populations were asked to rate the previously generated list of behaviours on their importance in defining an ideally intelligent, academically intelligent, and everyday intelligent person, and on how characteristic each behaviour was of these people. Both demographics conceived intelligence as a three factor structure, however the structures were different. Experts held a prototypical definition of intelligence as including verbal intelligence, problem-solving ability, and practical intelligence while laypeople defined it as including practical problem-solving ability, verbal ability, and social competence. Sternberg et al. (1981) noted how the first two factors in both models reflected the constructs of fluid and crystallised intelligence as described in Cattell and Horn’s Gf–Gc Theory (Cattell, 1941, 1963; Cattell & Horn, 1978; Horn & Cattell, 1966), while the third factors seemed to describe cohort specific practical intelligences. Where Sternberg et al. (1981) implemented this methodology outside of any particular context, Buckley et al. (2019) adopted it with initial technology teacher education students in relation to their prototypical definition of intelligence in STEM. They also found intelligence to be prototypically defined as a three factor model inclusive of social competence and general competence factors, and a third factor they termed technological competence which they inferred as a cohort specific factor.

Much work indicates how people of different ages and experiential backgrounds, both within and between groups, hold varied implicit theories, or prototypical definitions, regarding the structure of intelligence (Fry, 1984; Leahy & Hunt, 1983; Mason & Rebok, 1984; Mugny & Carugati, 1989; Yussen & Kane, 1983). This is of importance as such definitions govern the way people evaluate the intelligence of others (Sternberg, 2000) which has direct relevance to how young people self-evaluate relative to what is considered of importance within a field, how people stereotype the intelligence (both its nature and structure) of others, how practitioners within a field discuss the characteristics associated with success within their field, and how these occurrences interact on an individual psychological level and on subsequent decision making. As such, understanding the prototypical definition of intelligence as it is associated with engineering is significant, particularly when integrated within the work associated with the field-specific abilities beliefs hypothesis. Understanding what characteristics denote field specific brilliance or intelligence within engineering may aid in providing insight into the gender gap. Should there be a cohort specific factor as was revealed in previous studies (Buckley et al., 2019; Sternberg et al., 1981), or multiple cohort specific factors within engineering, it would allow for the relationship between young girls self-concept and interest in pursuing engineering to be more explicitly explored.

## Method

### Approach

In order to identify and determine the importance of perceived factors of an intelligent engineer from the perspective of third level engineering students across Ireland and Sweden, a survey methodology was employed (Sternberg et al., 1981). Two surveys were administered consecutively to cohorts of third level engineering students (Bachelors and Masters level) in Ireland and Sweden. The first was designed to elicit the characteristics which Irish and Swedish engineering students perceived to describe an intelligent engineer. The second was designed to capture participants’ rating of importance of each characteristic as they related to their own conception of an intelligent engineer so as to address the remaining objectives associated with identified broad factors descriptive of intelligent engineers and explorations of rated differences between samples. Reflecting the prior work of (Leslie et al., 2015), engineering was considered holistically and therefore students from a variety of engineering fields were invited to participate voluntarily. Considering the variation between Ireland (14.33%) and Sweden (28.33%) in female representation in higher education engineering (OECD, 2020), a cultural effect was hypothesised to influence the perception of what is characteristic of an innately intelligent engineer. Therefore, male and female participants were included from Ireland and Sweden to test the gender × country interaction with regard to this.

In Ireland, the surveys were sent to students in two higher education institutions, one University and one Institute of Technology (IoT), to reflect the two types of providers of engineering education in the country. In the University each of the surveys were sent to approximately 600 students and in the IoT they were both sent to approximately 800 students. In Sweden, the surveys were sent to a random sample of 2000 students in the country’s largest university level engineering education provider. Ethical approval was granted for data collection in Ireland by the Athlone Institute of Technology research ethics committee and was not required for data collection in Sweden. At the beginning of each survey, participants were informed about the contents and purpose of the survey, that responses would be anonymous and that participation was voluntary, and that by completing the survey they were consenting for their responses to be analysed in alignment with the aims of the research.

### Survey 1

#### Participants

Overall, 336 students responded to the first survey. A total of 174 students from the Swedish University responded (*M_age_* = 20.810, *SD_age_* = 2.225), of which 122 were male, 50 were female, and 2 chose not to disclose their gender. A total of 162 students from the two Irish institutions responded to the survey (*M_age_* = 23.747, *SD_age_* = 7.433), 85 from the University and 77 from the IoT, which were considered as a single cohort. Of these students, 121 were male, 40 were female, and 1 chose not to disclose their gender. Demographic information regarding the participants who responded to the survey are presented in Table 1.

**Table 1.** Respondent demographic information for Survey 1.

Course	% of Cohort	n	Year of study (n)				
			1	2	3	4	5
***Swedish respondents***							
IT and computer technology	32.76	57 (41 male, 14 female, 2 prefer not to say)	31	24	2	–	–
Mechanical engineering, industrial technology and finance	29.89	52 (37 male, 15 female)	51	–	–	–	1
Architecture, built environment and construction technology	8.05	14 (8 male, 6 female)	7	–	1	–	–
Common entry programme	6.32	11 (8 male, 3 female)	11	–	–	–	–
Vehicle engineering	7.47	13 (12 male, 1 female)	13	–	–	–	–
Energy and environment	5.75	10 (3 male, 7 female)	10	–	–	–	–
Electrical engineering, engineering physics and applied mathematics	4.02	7 (7 male)	6	1	–	–	–
Design and product development	3.45	6 (3 male, 3 female)	6	–	–	–	–
Technology and learning	1.72	3 (2 male, 1 female)	–	3	–	–	–
Medical technology	.58	1 (1 male)	–	1	–	–	–
***Irish respondents***							
Mechanical engineering	18.52	30 (25 male, 4 female, 1 prefer not to say)	7	10	6	7	–
Civil engineering	17.90	29 (25 male, 4 female)	5	11	4	8	1
Software engineering	16.67	27 (13 male, 14 female)	8	5	3	10	1
Engineering management	13.58	22 (17 male, 5 female)	8	14	–	–	–
Electronics and computer engineering	9.26	15 (13 male, 2 female)	3	4	3	4	1
Industrial engineering	7.41	12 (11 male, 1 female)	11	–	1	–	–
Biomedical engineering	4.94	8 (1 male, 7 female)	5	3	–	–	–
Polymer engineering	4.32	7 (5 male, 2 female)	–	1	5	1	–
Quantity surveying	2.47	4 (4 male)	1	2	1	–	–
Product design engineering	1.85	3 (3 male)	–	–	3	–	–
Aeronautical engineering	1.24	2 (2 male)	–	2	–	–	–
Mechatronics engineering	1.24	2 (1 male, 1 female)	1	–	–	1	–
Electrical engineering	.62	1 (1 male)	–	1	–	–	–

#### Instrument and procedure

Following information regarding informed consent and the purpose of the study, participants were asked for demographic information associated with their current studies (engineering field, degree level, and year of study), their age and their identifying gender. They were then asked to list behaviours characteristic of intelligence in engineering. In this instance, the exact wording was “Please list all of the characteristics or qualities of a person you would describe as intelligent in the context of engineering”. The surveys were administered electronically to students individually via their institutional email accounts on the 14th of February 2019. The Irish participants received and responded to the surveys in English while the Swedish students received and responded to the surveys in Swedish.

#### Data analysis

All data analysis was conducted in English, with all translations being done by a native Swede who was fluent in both English and Swedish to ensure for accuracy in translations with regard to the intended meaning on surveys and responses. The first stage of the analysis involved coding each of the characteristics offered by participants. The list generated from the Swedish sample was initially coded with an inductive approach and subsequently the generated codes were used to deductively code the list generated by the Irish sample. A list of 683 characteristics (*M* = 3.96, *SD* = 3.13) was generated from the Swedish participants. A total of 436 remained once literal duplicates were removed. The characteristics were primarily coded by two members of the research team. Initially one researcher coded all of the data by manually collating each of the 436 characteristics into groups based on the similarity of their wording, which resulted in a total of 80 unique codes being created. For example, the statements “good ability to think abstract” and “able to abstract problems so that they become more gripable” were coded as ‘abstraction’, while the statements “creative thinking” and “easy to get many quick ideas others would call creative” were coded as ‘creativity’. A second researcher then reviewed each of the codes and commented on their uniqueness within the list. At this stage, the second researcher identified eight of the codes, i.e., four pairs, as synonyms. These were reviewed collectively by both researchers and four codes were revised to clarify their distinctions. For the second stage, the first researcher reviewed each of the characteristics they had coded again based on the revisions to the codes while the second researcher independently coded each of the 436 characteristics using the established coding scheme. When compared, there were 17 discrepancies indicating a 96.10% level of agreement between the researchers when applying the codes. Finally, a third member of the research team coded each of the 17 discrepancies to aid in assigning their final codes.

**Figure 1.**
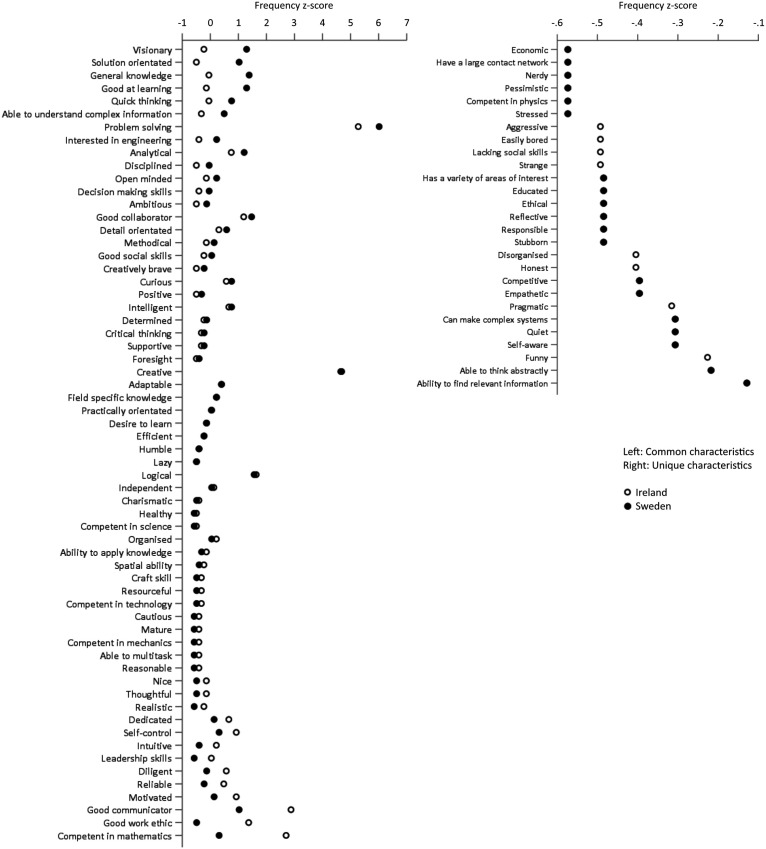
Frequencies of codes based on the responses to survey 1. Codes which appeared in the responses from students from both countries (left) are organised based on *z*-score differences. Codes which appeared only in responses from one of the countries (right) are organised based on *z*-score values. Vertical axes represent codes. Horizontal axes are presented at different scales.

A similar process was conducted with the data from the Irish sample. A list of 619 characteristics *(M* = 3.80, *SD* = 1.91) were generated, with 340 remaining once literal duplicates were removed. Both the first and second researcher independently applied the coding scheme generated from the Swedish data to the list of 340 characteristics. When compared there were six discrepancies, indicating a 98.24% level of agreement. Both researchers agreed that there were 15 characteristics for which existing codes did not suffice. They collectively created 9 new codes, which the third researcher reviewed and confirmed. Therefore, a total of 89 unique codes, representing 89 unique perceived characteristics of an intelligent engineer, were generated from the survey results. A list of all codes can be found in Figure 1, and a full codebook with example statements for each code can be found in Table S1 (Supplementary Material 1).

#### Results

The frequencies of each codes were considered relative to the complete lists of 683 characteristics from the Swedish sample and 619 from the Irish sample. In order to compare the frequencies of codes from across both samples’ the frequencies were converted to *z*-scores. As both lists were independent of each other and had different means and standard deviations as presented in the previous section, these were transformed to have identical means (*x̄* = 0) and standard deviations (*σ* = 15). Figure 1 illustrates the frequencies of codes that were common and unique to each sample in terms of *z*-scores.

#### Discussion

Considering Neisser’s (1979) description of the prototype of a category or concept as being that which displays all the typical properties, code frequency can be interpreted as an indication of the prototypical definitions of intelligent engineers held by the participants. Codes with high frequencies reflect characteristics cited more typically than those with lower frequencies. With being a good communicator, having a good work ethic and being competent in mathematics identified more frequently by the Irish participants and with being visionary, solution orientated and having general knowledge identified more frequently by the Swedish participants, these are indicative of differences in the typical properties perceived to reflect intelligent engineers between each sample. The existence of codes unique to each country, albeit in relatively low frequencies (*z* < -1), further suggests merit in exploring perceptions of intelligence at a country level. Also of interest are the select codes that appear in high frequencies across both samples. Problem solving and creativity were, relatively speaking, identified very frequently (*z* > 3) in both the Irish and Swedish samples as being characteristic an intelligent engineer, suggesting that these in particular were central to the participants’ prototypical definitions and are perhaps generalisable to other populations.

While being able to compare relative frequencies is an advantage of this survey with respect to identifying typical characteristics, there is an inherent limitation in that frequencies seldom provide a direct indication of which characteristics are perceived as more important to the participants’ conception of an intelligent engineer. Furthermore, it is possible that for individual participants there were characteristics of intelligent engineers which they did not immediately associate at the time they were responding to the survey. In response to these limitations, to permit the identification of broad factors descriptive of intelligent engineers, and to enable explorations into differences in their perceived importance between samples, a second survey was administered requiring participants to rate the importance of each code with respect to their conception of an intelligent engineer.

### Survey 2

#### Participants

Overall, 362 students responded to the second survey. A total of 190 students from the Swedish university responded (*M_age_* = 20.889, *SD_age_* = 2.308), of which 126 were male, 63 were female, and 1 chose not to disclose their gender. A total of 172 students from the two Irish institutions responded to the survey (*M_age_* = 22.535, *SD_age_* = 7.006), 102 from the University and 70 from the IoT, again considered as a single cohort. Of these students, 141 were male, 28 were female, and 3 chose not to disclose their gender. Demographic information regarding the participants who responded to the survey are presented in Table 2.

**Table 2.** Respondent demographic information for Survey 2.

Course	% of Cohort	n	Year of study (n)							
			1	2	3	4	5	6	7	
***Swedish respondents***										
Mechanical engineering, industrial technology and finance	32.63	62 (42 male, 20 female)	61	–	–	–	1	–	–	
IT and computer technology	31.58	60 (50 male, 9 female, 1 prefer not to say)	39	18	2	–	1	–	–	
Architecture, built environment and construction technology	10.53	20 (8 male, 12 female)	19	–	–	–	1	–	–	
Energy and Environment	7.90	15 (4 male, 11 female)	15	–	–	–	–	–	–	
Vehicle engineering	4.21	8 (7 male, 1 female)	7	–	–	–	–	–	1	
Technology and learning	3.68	7 (4 male, 3 female)	1	6	–	–	–	–	–	
Media technology	3.16	6 (4 male, 2 female)	–	6	–	–	–	–	–	
Common entry programme	2.63	5 (4 male, 1 female)	4	–	–	–	1	–	–	
Electrical engineering, engineering physics and applied mathematics	1.58	3 (3 male)	3	–	–	–	–	–	–	
Design and product development	1.58	3 (3 female)	3	–	–	–	–	–	–	
Chemistry and biotechnology	.53	1 (1 female)	–	1	–	–	–	–	–	
***Irish respondents***										
Mechanical engineering	29.65	51 (45 male, 6 female)	41	–	10	–	–	–	–	
Engineering management	26.16	45 (38 male, 7 female)	17	23	–	5	–	–	–	
Mechatronics engineering	10.47	18 (17 male, 1 female)	7	1	10	–	–	–	–	
Aeronautical engineering	9.30	16 (11 male, 4 female, 1 prefer not to say)	16	–	–	–	–	–	–	
Software engineering	5.81	10 (5 male, 4 female, 1 prefer not to say)	4	–	3	1	2	–	–	
Polymer engineering	5.23	9 (7 male, 2 female)	8	–	1	–	–	–	–	
Design and manufacture engineering	4.07	7 (6 male, 1 prefer not to say)	4	2	–	1	–	–	–	
Industrial engineering	3.49	6 (6 male)	4	2	–	–	–	–	–	
Biomedical engineering	2.91	5 (1 male, 4 female)	4	1	–	–	–	–	–	
Robotics and automation	1.74	3 (3 male)	3	–	–	–	–	–	–	
Materials design and engineering	.58	1 (1 male)	1	–	–	–	–	–	–	
Medical engineering	.58	1 (1 male)	1	–	–	–	–	–	–	

Notably, variances in gender representation across the fields of study of respondents and in the sample sizes between fields presented a limitation in terms of comparing the data between countries. Some engineering fields are also more heavily represented in the data, and an interaction with engineering field cannot be ruled out but was not examined due to a lack of adequate representation in a number of fields.

#### Instrument and procedure

For the second survey, participants were provided with the same informed consent information and asked the same demographic questions, however the second part of the survey contained a list of each unique characteristic (n = 89) mentioned in the responses to the first survey (see Figure 1 and Table S1), with participants being asked to rank each one on a 5-point Likert scale with the ratings “1 - Not important at all”, “2 – Unimportant”, “3 - Neither important nor unimportant”, “4 – Important”, and “5 - Very important” (Cohen et al., 2007). In this instance, the exact wording used was “please rate how important each of these characteristics are in defining ‘your’ conception/understanding of an intelligent engineer”. The surveys were administered electronically to students individually via their institutional email accounts on the 24th of April 2019. The Irish participants received and responded to the surveys in English while the Swedish students received and responded to the surveys in Swedish.

#### Data analysis

All data analysis was conducted in English, with all translations being done by a native Swede who was fluent in both English and Swedish to ensure for accuracy in translations with regard to the intended meaning on surveys and responses. The first stage of the analysis involved the conduction of an EFA with all responses to the second survey to identify broad factors perceived to reflect an intelligent engineer based on both samples. These factors were then examined to determine which were rated most important to the participants’ conceptions of an intelligent engineer. This was examined with respect to all participants as a single cohort, and a gender × country interaction was then subsequently tested. Due to violations in assumptions of normality, non-parametric tests were used. Following this, as there were characteristics identified uniquely in by participants from each country, EFA solutions were explored individually for the Irish and Swedish samples to see if they aligned with the consolidated EFA solution.

#### Results

**Figure 2.**
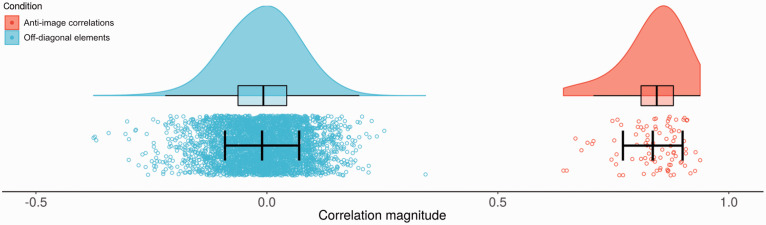
Anti-image correlations and off-diagonal elements. Boxplots represent quartiles. Means ± 1 standard deviation are displayed within data points.

To determine the factorability of the dataset for an EFA, the correlation matrix, anti-image correlation matrix, Kaiser-Meyer-Olkin (KMO) measure of sampling adequacy, and Bartlett’s test of sphericity statistic were examined (Tabachnick & Fidell, 2007). Of the 3916 correlations in the correlation matrix, 439 were greater than or equal to .3. An examination of the anti-image correlation matrix revealed that all anti-image correlations were above .5, and the off-diagonal elements were mostly small (Figure 2). The KMO measure of sampling adequacy was .852, above the recommended value of .6 (Kaiser, 1974), and Bartlett’s test of sphericity was significant (χ^2^ (3916) = 12668.621, *p* < .001). Therefore, there was a reasonable level of factorability within the data.

**Figure 3.**
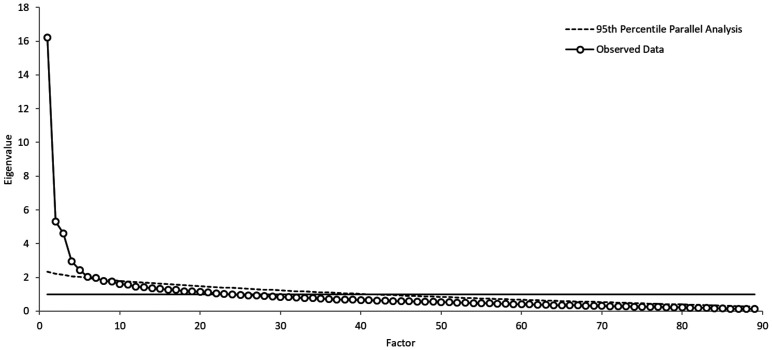
Factor eigenvalues and parallel analysis to determine number of EFA factors to extract.

A number of suggested criteria exist to determine the numbers of factors to extract in an EFA analysis including basing the decision on factors with eigenvalues great than one (Kaiser, 1960), examining a scree plot (Cattell, 1966), and conducting a parallel analysis (Horn, 1965). The results of each of these approaches are presented in Figure 3. A total of 24 factors had eigenvalues greater than 1, the scree plot suggests potential five or seven factor solutions, and the parallel analysis suggests a seven factor solution. As Horn’s parallel analysis has been identified as one of the most accurate a priori empirical criteria with scree sometimes a useful addition (Velicer et al., 2000), it was decided to extract seven factors during the EFA analysis.

The results of the seven factor solution are presented in Table 3. Characteristics with high salient pattern coefficients (pattern coefficients greater than .4 and less than -.4) are presented in bold text, and were the only characteristics used in the theoretical interpretation of factor meanings. For example, factor 1 (F1) was interpreted based on the 17 characteristics with high pattern coefficients associated with it listed in Table 3.

**Table 3.** Seven factor oblique EFA solution.

Characteristic	F1	F2	F3	F4	F5	F6	F7	*h^2^*
Creatively brave	**.694** (.652)	−.079 (.212)	.168 (.381)	−.138 (.277)	−.209 (−.116)	.056 (.286)	.089 (.139)	.608
Craft skill	**.630** (.663)	−.015 (.251)	−.073 (.305)	.032 (.426)	−.013 (.043)	.185 (.406)	.158 (.203)	.652
Intuitive	**.568** (.585)	−.115 (.147)	.118 (.329)	.017 (.355)	.029 (.116)	−.035 (.157)	.043 (.041)	.516
Quick thinking	**.559** (.550)	−.242 (.059)	.024 (.266)	−.007 (.363)	.236 (.261)	.117 (.220)	.029 (.022)	.600
Able to think abstractly	**.531** (.421)	−.035 (.080)	−.201 (.057)	−.036 (.223)	.113 (.111)	−.009 (.086)	.080 (.019)	.494
Creative	**.490** (.473)	−.004 (.209)	.051 (.298)	−.049 (.247)	.092 (.153)	−.079 (.130)	.195 (.171)	.492
Leadership skills	**.485** (.562)	.041 (.341)	.248 (.485)	−.106 (.262)	.042 (.165)	.017 (.241)	.052 (.107)	.660
Resourceful	**.460** (.612)	.268 (.449)	.106 (.397)	.017 (.329)	−.079 (.091)	.038 (.233)	−.211 (−.133)	.566
Decision making skills	**.445** (.402)	−.008 (.218)	.207 (.343)	−.210 (.092)	.052 (.129)	−.072 (.080)	.050 (.055)	.430
Able to multitask	**.431** (.498)	−.055 (.185)	.177 (.351)	.023 (.307)	.051 (.153)	−.047 (.131)	.003 (.017)	.496
Practically orientated	**.430** (.591)	.172 (.370)	−.081 (.308)	.121 (.432)	.073 (.171)	.195 (.369)	−.049 (.022)	.621
Stubborn	−**.419** (−.229)	−.070 (.066)	.241 (.200)	−.044 (−.074)	.260 (.233)	.317 (.261)	.090 (.216)	.486
Has foresight	**.415** (.521)	.103 (.262)	−.045 (.253)	.137 (.387)	.035 (.128)	.028 (.211)	.003 (.022)	.437
Charismatic	**.407** (.528)	.186 (.428)	.165 (.467)	−.029 (.280)	−.104 (.019)	.040 (.344)	.233 (.312)	.573
Competitive	**.406** (.509)	−.264 (.114)	.359 (.473)	−.031 (.322)	−.030 (.053)	.182 (.359)	.087 (.187)	.554
Spatial ability	**.406** (.498)	.094 (.274)	−.050 (.273)	.099 (.364)	.184 (.261)	−.001 (.180)	.064 (.059)	.541
Ethical	.104 (.178)	**.757** (.579)	−.366 (.035)	.044 (.053)	−.007 (.080)	−.167 (.028)	.108 (.082)	.581
Empathetic	−.002 (.050)	**.591** (.550)	−.133 (.179)	−.182 (−.116)	.081 (.127)	−.010 (.158)	.255 (.288)	.550
Honest	−.056 (.243)	**.591** (.580)	.007 (.302)	.172 (.208)	−.072 (.087)	.004 (.221)	−.017 (.081)	.566
Humble	−.175 (.061)	**.539** (.551)	.036 (.296)	.004 (.026)	−.047 (.046)	.117 (.315)	.210 (.334)	.578
Supportive	.006 (.375)	**.524** (.668)	.336 (.578)	.095 (.239)	−.164 (.068)	.007 (.307)	−.023 (.140)	.680
Nice	−.289 (.027)	**.518** (.570)	.207 (.406)	.041 (.037)	.022 (.151)	.051 (.264)	.180 (.313)	.638
Thoughtful	−.134 (.092)	**.491** (.525)	−.015 (.264)	−.028 (.042)	.313 (.399)	.111 (.176)	−.090 (−.012)	.594
Open minded	.011 (.188)	**.486** (.512)	.06 (.307)	.003 (.078)	.095 (.221)	−.117 (.075)	.057 (.092)	.478
Self-control	.057 (.296)	**.461** (.613)	.148 (.450)	−.099 (.107)	.175 (.313)	.143 (.291)	−.090 (.034)	.650
Reliable	.136 (.402)	**.434** (.546)	.225 (.463)	.109 (.257)	.007 (.215)	−.167 (.089)	−.082 (−.022)	.597
Has social skills	.216 (.350)	**.421** (.541)	.101 (.396)	−.088 (.118)	.035 (.166)	−.048 (.186)	.095 (.152)	.579
Ambitious	−.111 (.201)	−.041 (.268)	**.671** (.620)	.021 (.161)	−.011 (.154)	.044 (.228)	.007 (.161)	.512
Good work ethic	.204 (.430)	−.017 (.298)	**.543** (.584)	−.026 (.238)	.003 (.186)	.004 (.174)	−.175 (−.061)	.624
Positive	.011 (.290)	.237 (.479)	**.533** (.620)	−.049 (.126)	−.113 (.078)	−.004 (.255)	.058 (.208)	.626
Motivated	.069 (.298)	.077 (.311)	**.516** (.534)	.008 (.169)	−.050 (.126)	−.095 (.111)	−.035 (.059)	.518
Determined	.005 (.267)	−.047 (.239)	**.481** (.531)	.084 (.244)	.088 (.221)	.005 (.204)	.095 (.192)	.535
Competence in physics	−.168 (.314)	.082 (.134)	−.005 (.191)	**.771** (.701)	.022 (.161)	.055 (.228)	−.087 (−.016)	.618
Competence in mathematics	−.070 (.287)	−.142 (−.056)	.081 (.162)	**.700** (.642)	.055 (.165)	−.113 (.054)	−.002 (−.004)	.573
Competence in science	−.113 (.328)	.084 (.201)	.031 (.281)	**.649** (.647)	.178 (.304)	.018 (.230)	.066 (.115)	.661
Competence in mechanics	.202 (.552)	.010 (.155)	.006 (.269)	**.620** (.729)	.016 (.164)	−.031 (.204)	−.041 (−.014)	.699
Educated	.012 (.309)	.030 (.129)	.059 (.222)	**.464** (.493)	.038 (.147)	−.024 (.151)	.019 (.052)	.478
Competence in technology	.129 (.424)	.077 (.213)	−.039 (.252)	**.455** (.566)	.260 (.368)	−.023 (.152)	−.014 (−.010)	.563
Reflective	−.052 (.066)	.298 (.397)	−.042 (.242)	−.099 (.018)	**.550** (.573)	.043 (.100)	.100 (.104)	.564
Able to understand complex information	.014 (.278)	−.047 (.121)	−.002 (.221)	.347 (.450)	**.526** (.586)	.039 (.090)	−.096 (−.105)	.584
Solution orientated	.045 (.134)	.043 (.183)	.094 (.236)	−.011 (.107)	**.473** (.514)	−.094 (−.042)	.016 (−.021)	.532
Reasonable	.000 (.169)	.284 (.407)	−.016 (.275)	−.037 (.112)	**.461** (.514)	.113 (.170)	−.024 (.016)	.593
Field specific knowledge	−.043 (.020)	−.007 (.026)	−.088 (.030)	.137 (.137)	**.460** (.451)	−.123 (−.115)	.061 (−.021)	.488
Critical thinking	.278 (.253)	.145 (.231)	−.184 (.124)	−.027 (.149)	**.459** (.465)	−.137 (−.031)	.166 (.070)	.468
Problem solving	.110 (.103)	.010 (.072)	−.021 (.093)	.015 (.085)	**.412** (.427)	−.224 (−.177)	.063 (−.039)	.449
Methodical	.089 (.287)	.045 (.232)	.100 (.32)	.168 (.316)	**.412** (.491)	−.052 (.080)	.050 (.043)	.543
Lazy	.025 (.106)	−.075 (.057)	−.157 (.014)	−.061 (.102)	−.025 (−.101)	**.721** (.611)	−.079 (.110)	.587
Lacking social skills	.220 (.336)	−.063 (.174)	.037 (.226)	−.097 (.191)	−.092 (−.083)	**.629** (.611)	−.150 (.054)	.578
Stressed	.061 (.244)	−.030 (.181)	.047 (.231)	.010 (.214)	−.079 (−.070)	**.592** (.613)	−.025 (.179)	.599
Easily bored	.131 (.213)	−.053 (.124)	−.092 (.12)	−.061 (.154)	.019 (−.021)	**.579** (.553)	.000 (.156)	.536
Disorganised	.123 (.197)	.029 (.181)	−.074 (.123)	−.126 (.086)	−.023 (−.047)	**.574** (.531)	−.085 (.084)	.435
Strange	.093 (.302)	.055 (.242)	.006 (.252)	.103 (.291)	−.181 (−.153)	**.515** (.637)	.127 (.321)	.572
Pessimistic	−.176 (.089)	.018 (.155)	.089 (.205)	.158 (.212)	−.077 (−.054)	**.465** (.507)	.036 (.223)	.475
Has a variety of areas of interest	**.443** (.453)	.099 (.277)	−.145 (.242)	.033 (.304)	.006 (.023)	.073 (.354)	**.477** (.485)	.650
Curious	−.045 (.025)	.141 (.227)	.069 (.227)	−.009 (.032)	.166 (.181)	−.095 (.098)	**.446** (.438)	.473
α	.846	.838	.709	.806	.736	.791	.310	
Eigenvalue	16.232	5.322	4.611	2.962	2.435	2.035	1.987	
% of Variance	18.239	5.980	5.181	3.328	2.736	2.286	2.233	
Factor correlations								
F1	–							
F2	.350	–						
F3	.452	.495	–					
F4	.566	.134	.293	–				
F5	.139	.225	.279	.182	–			
F6	.299	.312	.330	.296	−.017	–		
F7	<.001	.137	.215	.057	−.066	.325	–	

Factor pattern coefficients (structure coefficients) based on maximum likelihood extraction with promax rotation (k = 4). Salient pattern coefficients presented in bold (pattern coefficient > .4 and > −.4) only were used to calculate Cronbach’s α. h2 = communality.

The first factor (F1) is most strongly loaded on by the characteristics of being creatively brave, intuitive, and quick thinking, as well as having good craft skill and being able to think abstractly. These appear to represent practical problem solving, i.e. being able to intuitively or quickly think of a solution which may be novel and being able to implement it. Many of the other items which load on this factor (having leadership and decision making skills, being resourceful, able to multitask, practically orientated, and having foresight) appear to reflect a leadership factor. Given the existence of much research linking gender and leadership, the exploration of a female leadership self-concept relative to the engagement of women with engineering has much merit. This is particularly important due to leadership traits being generally stereotyped as masculine, and the devaluing of women in leadership positions (Eagly, 2007; Eagly et al., 1992; Koenig et al., 2011).

The second factor (F2) is most strongly loaded on by the characteristics of being ethical, empathetic, honest, humble, supportive, and nice. Considering the other items which load of it, it appears to represent a factor describing conscientiousness.

The third factor (F3) is described by the characteristics of being ambitious, having a good work ethic, and being positive, motivated, and determined. It therefore appears to represent a factor describing drive.

The fourth factor (F4) is described by the characteristics of being competent in physics, mathematics, science, mechanics, and technology, as well as being educated. It therefore appears to represent a factor describing discipline knowledge.

The fifth factor (F5) is most strongly loaded on by the characteristics of being reflective and being able to understand complex information, and is also loaded on by the characteristics of being solution orientated, reasonable, methodical, having field specific knowledge, and being able to think critically and problem solve. It appears to represent a factor describing a way of thinking and a capacity to do so at great depth and/or about complex information. It will therefore be termed as ‘reasoning’.

The sixth factor (F6) is most strongly loaded on by the characteristics of being lazy, lacking social skills, being stressed, easily board, disorganized, strange, and pessimistic. There is a negative connotation associated with these characteristics relevant to most others, and it therefore appears to represent a factor describing negative attributes. However, this inference of a negative connotation is being subjectively applied, and subsequent analysis based on the Likert scale responses of the importance of the items loading on this factor may suggest otherwise.

The seventh factor (F7) is only loaded on by two characteristics, having a variety of areas of interest and being curious. With such little information it is difficult to infer a general factor with confidence, however it appears to represent a factor describing inquisitiveness. An important note of caution is that the factor has low reliability (α = .31) and the included characteristics correlate weakly (*r* = .193, *p* < .001). It’s inclusion in the EFA solution is based on the result of the parallel analysis suggesting a seven factor solution, so further inferences pertaining to the factor are limited.

**Figure 4.**
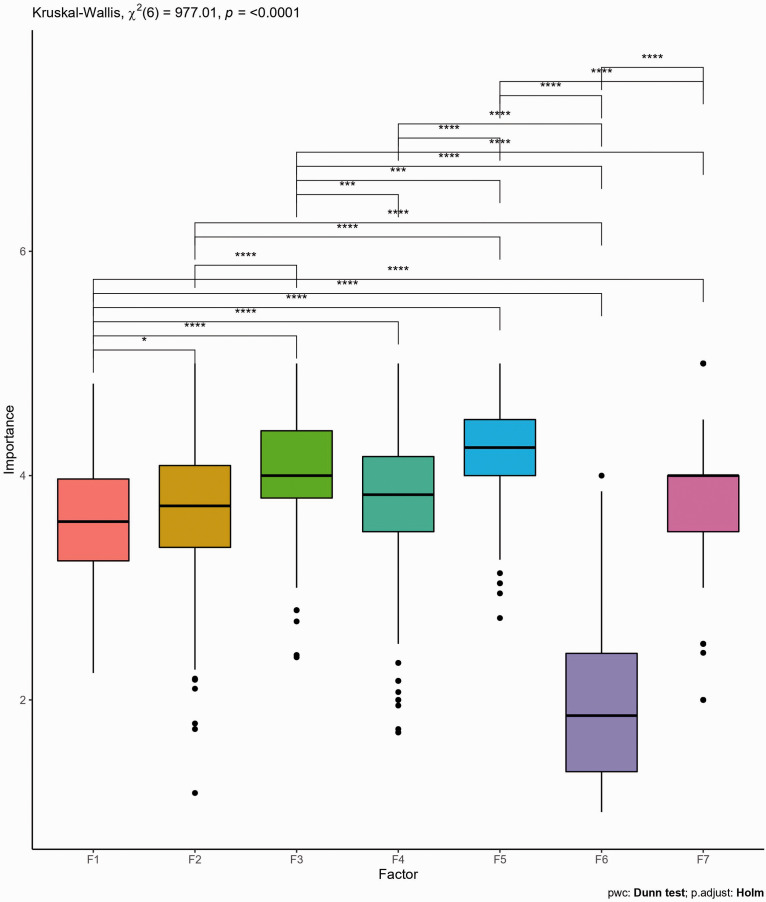
Perceived importance of each of the factors revealed by the EFA. Based on their interpretations, F1 = practical problem solving, F2 = conscientiousness, F3 = drive, F4 = discipline knowledge, F5 = reasoning, F6 = negative attributes, and F7 = inquisitiveness.

Subsequent to the EFA, the perceived importance of each factor was examined in terms of prototypically defining an intelligent engineer. In treating the seven factors, which represent factors of perceived importance for an intelligent engineer, as dependant variables for examining differences in rated importance, only the characteristics with high salient loadings (pattern coefficients greater than .4 and less than -.4) on each factor were considered. Hence, Factors 1 to 7 (practical problem solving, conscientiousness, drive, discipline knowledge, reasoning, negative attributes, and inquisitiveness) as presented in Figure 4 were derived from averaging participants’ scores on the characteristics that strongly loaded in each factor in the EFA result presented in Table 3. For example, Factor 2 (termed conscientiousness) is a thematic code derived from averaging participants’ scores on the 11 characteristics of an intelligent engineer that heavily loaded on the second EFA factor (ethical, empathetic, honest, humble, supportive, nice etc.; see Table 3 for the list). Items with a negative pattern coefficient, i.e., “stubborn” which loaded on factor 1, were reverse scored.

Prior to testing differences in reported importance between factors, several assumptions were tested. First, the data was screened for univariate outliers. These were identified as values below Q1 – (3 × IQR) or above Q3 + (3 × IQR) within each factor, where Q1 and Q3 represent the first and third quartiles respectively and IQR is the inter-quartile range (Q3 – Q1). Under these criteria there was one univariate outlier in Factor 2 ‘Conscientiousness’ with a score of 1.12 which was transformed to 1.17, the lower limit for univariate outliers in Factor 2, and there was one univariate outlier in Factor 7 ‘Inquisitiveness’ with a score of 1.92 which was transformed to 2, the lower limit for univariate outliers in Factor 7. Next, the assumption of homogeneity of variances was examined with a Levene test. The result was statistically significant, *F*(6) = 16.64, *p* < .001, indicating that the assumption was violated. Finally, a Shapiro-Wilk test was used to test the assumption of normality of residuals. The result was statistically significant, *W* = .99, *p* < .001, indicating the assumption was violated. As the data violated the assumptions of normality and equality of variances, the non-parametric Kruskal-Wallis test was used to compare rating of importance between each factor. The result was statistically significant, *χ*^2^(6) = 977.009, *p* < .001, and therefore post hoc testing was conducted with Dunn’s tests to compare mean rank sums. The Holm-Bonferroni method was used to control family-wise error rates. The results (Figure 4) indicate all factors but Factor 6 ‘Negative attributes’ were viewed as important to the participants conceptions of an intelligent engineer, with Factor 5 ‘Reasoning’ and Factor 3 ‘Drive’ being rated as most important. Statistically significant pairwise differences were observed between all factors except for between factors 2 and 4, factors 2 and 7, and factors 4 and 7.

The next stage of the analysis involved examining the potential gender × country interaction in the rated importance of each of the seven factors. First, participants who chose not to disclose their gender (n = 4) were removed from the dataset. To determine the appropriate statistical test, a number of assumptions were tested. Initially outliers were screened as previously indicated^1^. The assumption of univariate normality was checked for each of the four groups, i.e., Irish males, Irish females, Swedish males, and Swedish females, across each of the seven factors with Shapiro-Wilk tests. The assumption of normality was violated in 14 of the 28 instances (for test statistics see Table S2 in Supplementary Material 2). Next, a Shapiro-Wilk test for multivariate normality was conducted. The result was statistically significant, *W* = .976, *p* < .05, indicating that the assumption of multivariate normality was violated.

**Figure 5.**
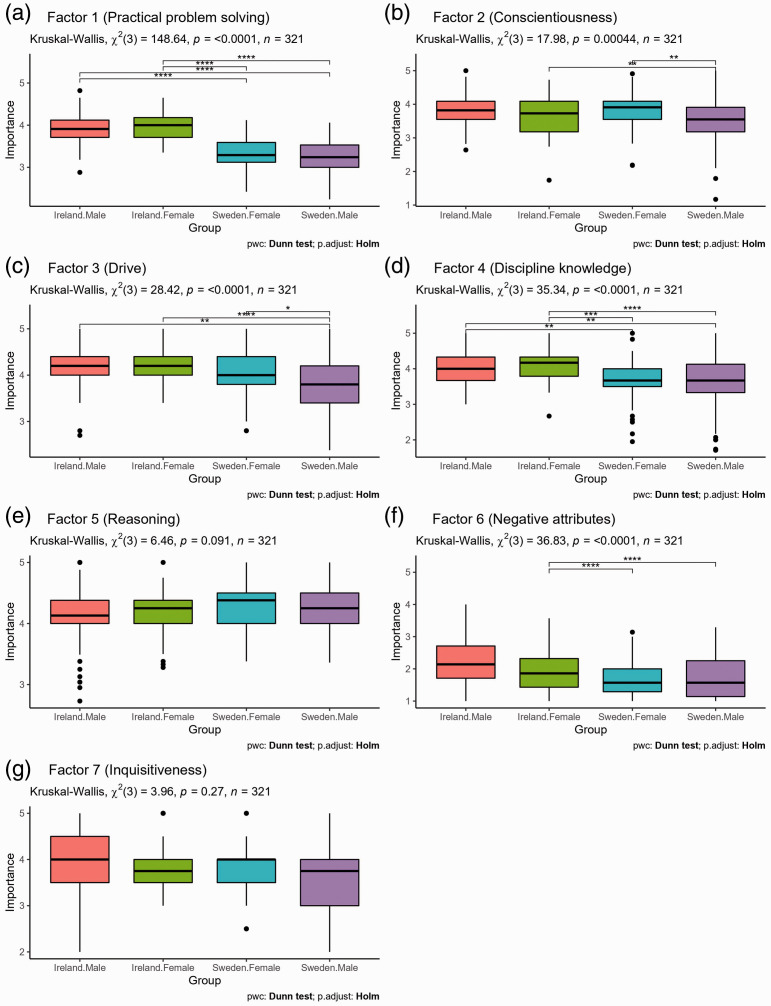
Pairwise comparisons of rated importance of EFA factors by participant country and gender.

As a result of violations to both univariate and multivariate normality, a non-parametric test of one-way multivariate data was performed using the npmv R package (Ellis et al., 2017). The package compares the multivariate distributions of the different samples by using F-approximations for ANOVA Type, Wilk’s Lambda Type, Lawley Hotelling Type, and Bartlett Nanda Pillai Type test statics, and conducts a permutation test for each. Using 1000 permutations, considering each of the seven factors as dependent variables and a gender × country interaction as the independent variable, a statistically significant result was observed, *F_approx._*(11.31, 829.25) = 10.566, *p* < .001, Wilk’s Λ = 18.505 in the non-parametric multivariate test. Based on this result, follow up non-parametric Kruskal-Wallis tests were conducted to test a gender × country interactions for each of the seven factors. Statistically significant gender × country interactions were observed for Factor 1 ‘Practical problem solving’, Factor 2 ‘Conscientiousness’, Factor 3 ‘Drive’, Factor 4 ‘Discipline knowledge’, and Factor 6 ‘Negative attributes’. The results of these, with post-hoc Dunn’s tests (Holm-Bonferroni correction) to compare differences across each group are presented in Figure 5. As a significant gender × country interaction effect was not found for Factor 5 ‘Reasoning’ and Factor 7 ‘Inquisitiveness’, main effects of gender and country were examined for these factors. A main effect of the participants’ country was found for Factor 5 ‘Reasoning’, *χ^2^*(1) = 5.018, *p* = 0.025 with the Swedish participants rating it higher (Median [MED] = 4.38, Median absolute deviation [MAD] = .37) than the Irish participants (MED = 4.19, MAD = .28). No significant effects were found for Factor 7 ‘Inquisitiveness’ which may be a result of poor suitability as an extracted factor.

As there were characteristics of intelligent engineers reported in Survey 1 which were unique to each country, it was of interest to check whether the 7 factor structure used in the prior analysis would hold if the Irish and Swedish sample were analysed separately. Full details of these analyses can be found in Supplementary Material 3.

The five factors of practical problem solving, conscientiousness, drive, discipline knowledge and negative attributes demonstrated degrees of consistency across each of the three EFA solutions. Considering the low importance ratings for Factor 6 ‘Negative attributes’ (Figure 4), it appears most accurate to infer that the prototypical definition of an intelligent engineer for the participants in this study is best described by the four factors of practical problem solving, conscientiousness, drive and discipline knowledge. The post-hoc pairwise comparisons for these factors (Figure 5) can be used to determine which factors are more central for group level definitions, however based on Neisser’s (1979) description of the prototype of a concept, it is the identification of the typical factors which is of most importance.

#### Discussion

Of the five factors which were apparent across each EFA structure (practical problem solving, conscientiousness, drive, discipline knowledge and negative attributes) statistically significant differences between Irish and Swedish females were found only for practical problem solving, discipline knowledge, and negative attributes. The biggest difference was observed in the ratings of importance for the practical problem solving factor, which was rated as more important by Irish females in comparison to Swedish females. Irish females also rated disciplinary knowledge as more important to their conception of an intelligent engineer than Swedish females but the difference in magnitude was smaller. It is possible that perceived relevance of practical problem solving and discipline knowledge act as barriers to Irish female engagement with engineering which is discussed further in the next section on cultural differences between Ireland and Sweden. There was also a statistically significant difference in the rated importance of the negative attributes factor, with Swedish females rating it as being less important to their conceptions of an intelligent engineer than Irish females. However, as neither group rated this factor as being important to their conceptions of intelligent engineers in general, it is unlikely that this factor has much practical significance.

The factors relating to consciousness and drive are also of particular relevance even though there were not rated significantly differently by Irish and Swedish females. The consciousness factor could be interpreted as general and it aligns with the social competence factors found by Buckley et al. (2019) and Sternberg et al. (1981). Based on this, while perceived by the participants to be relevant to intelligence in engineering, it is likely to be relevant to peoples’ conceptions of intelligence in general. The factor associated with drive is of particular interest in light of the field-specific ability beliefs hypothesis. Under the hypothesis, due to their being a large difference in female representation in higher education engineering education between Ireland and Sweden, it would be logical to hypothesise that Swedish females would associate engineering with drive (as a proxy for effort) to a greater degree than Irish females. However, no such statistical difference was observed. In fact, Irish females rated drive as being more important to their understanding of an intelligent engineer and the only significant differences associated with this factor were that Swedish males rated drive significantly less important than the other three groups.

## General discussion

### Cultural differences between Ireland and Sweden

Sweden is one of the worlds’ most egalitarian countries (Sendén et al., 2019) and is often seen to be a secularized society in comparison to other Western European countries. Ireland, in contrast, is viewed as less secularized, where Catholic traditions have been noted as one of the reasons why gender orders are still stronger and more hierarchical in comparison to the Swedish context (Inglehart & Welzel, 2005; O’Connor & Goransson, 2015). Sweden has also long had a political agenda to achieve a gender neutral labour market. This has been successful in the sense that relatively more women are active in the labour market in Sweden compared to Ireland, but less successful in that a predominance of women still work in female-dominated occupations and men in male-dominated occupations. The more egalitarian culture of Sweden could be related to why there is a higher rate of female representation in traditionally male dominated fields in general, but when considering third level engineering specifically, differences in the compulsory school structure of both countries likely have significant influence.

In order to increase overall interest in technology, Sweden introduced Technology as a compulsory school subject from pre-school to year 9 (pupil age ≈ 15) in 2010 (Skolverket, 2018). The intention was, among other things, that young people should be able to develop their relationship to the subject of Technology in a gender neutral way before making future career choices. There have been challenges in this agenda already at pre-school level. Hallström et al. (2015) for example found that Swedish girls and boys approach the Technology subject differently at pre-school which results in the confirmation rather than dissolution of gender boundaries, and Sandström et al. (2013) identified pre-school teachers as having different levels of understanding of gender related issues. In Ireland, technology education has a different structure. Technology does not feature as a unique subject area until secondary education, which pupils typically begin around the age of 12, and it is an optional area of study. Specifically, technology education in Ireland consists of four discrete school subjects at lower-secondary education (Wood Technology, Applied Technology, Engineering, and Graphics) and as four discrete subjects at upper-secondary level (Construction Studies, Technology, Engineering, and Design and Communication Graphics). Unlike in Sweden where Technology is a compulsory school subject, in Ireland females generally have less than 20% representation across the suite of optional technology subjects (Irish State Examinations Commission, 2019). A likely reason for the significant gender representation gap in technology education in Ireland is due to the subjects evolving from traditional technical education, which was either not accessible to young females or saw them having to study different topics to males within the same subject. The reason for highlighting differences in school systems as a potential reason for the results of this study goes beyond the more gender-balanced engagement with technology education in Sweden. In Ireland, as there is a school subject explicitly called ‘Engineering’ at secondary level, young people’s understanding of engineering as a discipline prior to third level is likely influenced by their knowledge of this. The subject has a substantial metal craft element (akin to practical problem solving), and a discrete body of disciplinary knowledge associated with performance as laid out by the relevant national curriculum. It is posited that these features are central to the group differences observed in rated importance between these factors (Figure 5). While the participants of this study, as third level students, would now have a greater understanding of engineering based on their third level education, the sample was heavily represented by students in their 1^st^ year of their courses so their conceptions were likely still influenced by their secondary level experience.

### The prototypical definition of an intelligent engineer

Beyond cultural explanations for the results, this work needs to be considered both in terms of the prototypical definition of an engineer, and in relation to the field-specific beliefs hypothesis with resulting implications for the gender disparity in engineering education. Unlike the previous studies by Sternberg et al. (1981) and Buckley et al. (2019), the results of this study indicated that the prototypical definition of an intelligent engineer held by Irish and Swedish engineering students reflects a five factor model with these factors appearing to represent practical problem solving, conscientiousness, drive, discipline knowledge, and negative attributes. However, two considerations must be taken into account when interpreting these factors. The factor termed ‘Negative attributes’ was qualitatively different than any other factor as each item with a positive salient loading on it had a negative connotation and relative to the other factors, it was not rated as being important to the participants conceptions of an intelligent engineer (see Figures 4 and 5). Therefore, it may be reasonable to interpret this factor as being perceived to be descriptive of people who are prototypically intelligent engineers, however it does not describe characteristics which are linked to what is perceived to make these people intelligent. Additionally, the first factor, termed ‘practical problem solving’ was a complex factor which seemed to have an additional leadership dimension. While this didn’t emerge as a standalone factor in any of the EFA’s, it is worth considering it in future confirmatory work in light of the research on gender and leadership.

Previous work studying prototypical definitions has revealed factors which were interpreted as cohort specific factors (Buckley et al., 2019; Sternberg et al., 1981). Identifying skills necessary for engineers is the focus of a substantial body of research (see Carthy et al., 2018, for a review) and identifying such cohort specific factors is related to this. Based on the four factors from this study interpretable as being associated with intelligence in engineering, i.e. not including the ‘Negative attributes’ factor due to low importance rating or the factors associated with reasoning or inquisitiveness as they weren’t apparent when the Irish and Swedish data were analysed independently, the ‘Conscientiousness’ factor could be interpreted as general, as could the ‘Drive’ factor which it has particular importance relative to the field-specific ability beliefs hypothesis. The ‘Practical problem solving’ factor could be interpreted as an engineering specific factor if the context of engineering is applied, and the ‘Discipline knowledge’ factor can also be interpreted as being engineering specific. Based on this, it appears that what the participants of this study consider to be the unique characteristics of an intelligent engineer, beyond their general intelligence and personality traits which could be generalised to denote intelligence in multiple contexts, is the knowledge an engineer embodies and their capacity to enact it in solving practical problems. This result, in conjunction with the previous studies examining prototypical intelligence (Buckley et al., 2019; Sternberg et al., 1981), further suggests merit in the use of this methodology for eliciting perceived factors describing cohort specific intelligence. Additionally, it adds a more nuanced perspective of what is meant, at least from the context of Irish and Swedish university students, by innate intelligence with respect to engineering and therefore has significant implications for future work exploring gender representation in engineering based with respect to the field-specific ability beliefs hypothesis.

### Field-specific ability beliefs in higher education engineering

Work associated with the field-specific ability beliefs hypothesis to date has explored conceptions of brilliance in disciplines holistically, such that denotations of exceptional ability included broad terms such as “brilliant” and “smart” (Bian et al., 2017; Leslie et al., 2015; Meyer et al., 2015). However, research associated with self-concepts suggests that while such broad self-ratings are important, it is also important to consider self-concepts of more specific attributes such as competency with mathematics (Sax et al., 2015). The results of this study provide a selection of characteristics associated with perceptions on an intelligent engineer to further this agenda. Future work should consider the self-concepts of young people in relation to the items with salient loadings on the cohort specific factors such as the knowledge domains of perceived importance both with and other than mathematics i.e., physics, science, mechanics, and technology, and the items associated with practical problem solving such as being creatively brave and having good craft skill. This would allow for investigation into how the self-concepts of young people effect their interest and engagement with higher education engineering, when the aspects of their self-concept they are reflecting on are perceived to be associated with either engineering specifically or are perceived to relate to a general understanding of intelligence.

Additionally, work to date on the field-specific ability beliefs hypothesis has been conducted exclusively in the USA (Bian et al., 2017; Leslie et al., 2015; Meyer et al., 2015). The results of this study indicate that the magnitude of factors perceived to describe intelligence, at least from the perspective of university engineering students in Ireland and Sweden, is influenced by cultural context and gender. This suggests that although the results associated with the field-specific ability beliefs hypothesis are robust (e.g., Cimpian & Leslie, 2015) they may not be generalisable beyond the USA, at least at the same magnitude. For example, the interest women and girls have in Sweden or other countries for engaging in engineering may or may not be influenced by practitioner’s field-specific beliefs to the same degree as those in the USA, and this may be due to variations in the prototypical definition of an intelligent engineer. So it is therefore important for future work associated with the field-specific ability beliefs hypothesis to consider the cultural context of participants, their gender, and their prototypical definitions of intelligence or brilliance, so as to determine how these may mediate people’s interests and associated gender disparities within certain fields.

## Conclusion and limitations

This paper offers a range of empirical insights associated with advancing engineering education, particularly in relation to field-specific ability beliefs with implications for research associated with engineering gender stereotypes. Perhaps of greatest importance is the evidence illustrating the need to consider the variance in prototypical definitions of intelligence across cohorts and cultures when considering self-concept and its association with field interest and engagement. In this study, culture was defined in terms of the country the participants were studying in and there is significant evidence which illustrates nation-level differences on factors associated with variables which influence engineering interest, engagement, and performance (Charles et al., 2014; Hamamura, 2012; Mann & DiPrete, 2016; Nosek et al., 2009; Reilly, 2012; Stoet et al., 2016). In line with this evidence and as hypothesised, this study illustrated a gender × country interaction with respect to the magnitude of importance given to specific factors perceived to denote intelligence in engineering. Considering evidence illustrating a difference in female students self-concepts (e.g., Blatchford, 1997; Langan et al., 2008; Torres-Guijarro & Bengoechea, 2017) and self-determined performance standards (Mann & DiPrete, 2016) with respect to male students, within the theoretical framework of the field-specific ability beliefs hypothesis, future work should consider potential variances between males and females with regards to their self-concepts and self-determined performance standards relative to the factors observed in this study. Additionally, future work should also explore potential manifestations of cultural differences between countries and how they relate to engagement in engineering. Finally, this study explored the perceptions of students within engineering education. As the overall aim is to make engineering more inclusive, there is a need for future work to consider the perceptions of underrepresented groups who select not to enter engineering. Such future work, if conducted within Ireland and Sweden, could use the dataset described within this paper as a comparative basis.

There are limitations with this study which should be acknowledged. The participants were university students predominantly pursuing bachelors and masters level degrees and therefore had a different level of experience to the graduate students and faculty who were the subject of previous work associated with the field-specific ability beliefs hypothesis. The samples are also not representative of all engineering fields and small sample sizes across fields prevented explorations of a possible interactions with the engineering fields. While it was intended to collect data from students across a broad array of engineering fields, the resulting samples have differences in representation across fields limiting comparability between the two countries. The same applies to gender representation. While it was anticipated that there would be fewer female participants in the Irish sample as a result of their being proportionately fewer female engineering students in the country, this discrepancy in gender representation also limits comparability of results across countries. The participants may also have been influenced by dominant philosophies within their institutions. However as there are large numbers of engineering faculty members in the involved institutions who will have impacted the student experience, without further empirical work it is not possible to verify if there was such an influence and to what extent it may have impacted the results. Finally, the factor interpreted as practical problem solving contained items which do not all qualitatively appear to align with a single factor. Many items related to leadership and in light of the research associating gender stereotypes and leadership (Eagly, 2007; Eagly et al., 1992; Koenig et al., 2011) so it would be useful for future confirmatory work to revisit this. While future work should acknowledge these limitations, the results provide new evidence to support investigations into the implicit and explicit barriers to women entering engineering based around gender and field biases and stereotypes.

## Data availability

The datasets generated during and/or analysed during the current study are available from the corresponding author on reasonable request.

## Supplemental Material

sj-pdf-1-prx-10.1177_00332941211000667 - Supplemental material for Exploring the Prototypical Definitions of Intelligent Engineers Held by Irish and Swedish Higher Education Engineering Students Click here for additional data file. Supplemental material, sj-pdf-1-prx-10.1177_00332941211000667 for Exploring the Prototypical Definitions of Intelligent Engineers Held by Irish and Swedish Higher Education Engineering Students by Jeffrey Buckley, Tomás Hyland, Lena Gumaelius, Niall Seery and Arnold Pears in Psychological Reports

sj-pdf-2-prx-10.1177_00332941211000667 - Supplemental material for Exploring the Prototypical Definitions of Intelligent Engineers Held by Irish and Swedish Higher Education Engineering Students Click here for additional data file. Supplemental material, sj-pdf-2-prx-10.1177_00332941211000667 for Exploring the Prototypical Definitions of Intelligent Engineers Held by Irish and Swedish Higher Education Engineering Students by Jeffrey Buckley, Tomás Hyland, Lena Gumaelius, Niall Seery and Arnold Pears in Psychological Reports

sj-pdf-3-prx-10.1177_00332941211000667 - Supplemental material for Exploring the Prototypical Definitions of Intelligent Engineers Held by Irish and Swedish Higher Education Engineering Students Click here for additional data file. Supplemental material, sj-pdf-3-prx-10.1177_00332941211000667 for Exploring the Prototypical Definitions of Intelligent Engineers Held by Irish and Swedish Higher Education Engineering Students by Jeffrey Buckley, Tomás Hyland, Lena Gumaelius, Niall Seery and Arnold Pears in Psychological Reports

## Note

1 One univariate outlier in Factor 2 ‘Conscientiousness’ with a score of 1.12 was transformed to 1.17, the lower limit for univariate outliers in Factor 2, and one univariate outlier in Factor 7 ‘Inquisitiveness’ with a score of 1.92 was transformed to 2, the lower limit for univariate outliers in Factor 7. There were an additional three multivariate outliers in the data, as assessed by Mahalanobis distance (*p* > 0.001). These were removed from the dataset.

## Notes

**Author Biographies:** **Jeffrey Buckley** is an assistant lecturer at Athlone Institute of Technology, Ireland, and an Affiliate Faculty Member of the Department of Learning at KTH, Royal Institute of Technology, Sweden. He is a member of both the Technology Education Research Group (TERG) and the Engineering Education in Society research group. Jeffrey received his PhD from KTH in 2018 in the area of spatial ability and learning in technology education. His current research interests include the relationship between spatial ability and learning, pedagogy and educational assessment, diversity and inclusivity in engineering education, and methods and practices in technology and engineering education research. **Tomás Hyland** is currently undertaking a PhD in the Faculty of Engineering and Informatics in Athlone Institute of Technology under the supervision of Dr Niall Seery, Dr Jeffrey Buckley and Dr Seamus Gordon. Tomás’ PhD research focuses on mainly on the areas of learning, cognitive load theory and spatial cognition. He has a particular interest in conducting school based research to gain insight into authentic classroom activity and learning. He is conducting research with both post-primary and university students looking specifically at if having elevated spatial skills reduces the cognitive load associated with learning new fundamental Engineering/Technology concepts. **Lena Gumaelius** is associate professor in Engineering Education at KTH, Royal Institute of Technology, Sweden, where she is leading the Engineering Education in Society research group. Her main research interest is in engineering and technology education, where she focuses on inclusiveness and attractiveness as well as education for sustainable development. Lena has a background as an Engineer, with a PhD in biotechnology, but changed research area as she became responsible for establishing Engineering Education at KTH. Currently, Lena holds the position as the Deputy Vice chancellor at Mälardalen University. **Niall Seery** is currently deputy president of Athlone Institute of Technology. He served as Vice President of Academic Affairs and Registrar before taking the role as Director of the Technological University Project at AIT. He has a PhD in Engineering Education and has a background in Engineering and Technology Teacher Education, where he spent 15 years as an academic with a specialist interest in pedagogical practice. In 2010, he founded and continues to direct the Technology Education Research Group. He has supervised a number of PhD students to completion and actively contributes to a number of journal editorial boards in the area of Design and Technology education. Niall also served as a visiting associate professor of Technology Education at the Royal Institute of Technology, KTH in Stockholm. **Arnold Pears** is foundation professor of Engineering Education and Chair of the Department of Learning in Engineering Sciences at KTH Royal Institute of Technology, Sweden. He has published over 100 articles in Engineering and Computing Education Research and is the IEEE Education Society Vice President for Publications 2020-2021.

## References

[bibr1-00332941211000667] Adams R. Fincher S. Pears A. Börstler J. Boustedt J. Dalenius P. Eken G. Heyer T. Jacobsson A. Lindberg V. Molin B. Moström J.-E. Wiggberg M. (2007). What is the word for “engineering” in Swedish: Swedish students’ conceptions of their discipline. Department of Information Technology.

[bibr2-00332941211000667] Bahník Š. Vranka M. A. (2017). Growth mindset is not associated with scholastic aptitude in a large sample of university applicants. Personality and Individual Differences, 117(1), 139–143. 10.1016/j.paid.2017.05.046

[bibr3-00332941211000667] Bian L. Leslie S.-J. Cimpian A. (2017). Gender stereotypes about intellectual ability emerge early and influence children’s interests. Science (New York, N.Y.), 355(6323), 389–391. 10.1126/science.aah6524 28126816

[bibr4-00332941211000667] Bian L. Leslie S.-J. Murphy M. Cimpian A. (2018). Messages about brilliance undermine women’s interest in educational and professional opportunities. Journal of Experimental Social Psychology, 76(1), 404–420. 10.1016/j.jesp.2017.11.006

[bibr5-00332941211000667] Blatchford P. (1997). Pupils’ self assessments of academic attainment at 7, 11 and 16 years: Effects of sex and ethnic group. British Journal of Educational Psychology, 67(2), 169–184. 10.1111/j.2044-8279.1997.tb01235.x. 9193172

[bibr6-00332941211000667] Borrego M. (2008). Creating a culture of assessment within an engineering academic department. In *38th ASEE/IEEE frontiers in education conference* (pp. 1–6). IEEE.

[bibr7-00332941211000667] Buckley J. O’Connor A. Seery N. Hyland T. Canty D. (2019). Implicit theories of intelligence in STEM education: Perspectives through the lens of technology education students. International Journal of Technology and Design Education, 29(1), 75–106. 10.1007/s10798-017-9438-8

[bibr8-00332941211000667] Carthy D. Bowe B. Gaughan K. (2018). *What are the engineering professional competences?* [Paper presentation]. 46th SEFI Conference (pp. 1–7), 18–21 September 2018, *European Society for Engineering Education*, Copenhagen, Denmark.

[bibr9-00332941211000667] Cattell R. (1941). Some theoretical issues in adult intelligence testing. Psychological Bulletin, 38(7), 592. https://doi.org/10.1037/h0050099

[bibr10-00332941211000667] Cattell R. (1963). Theory of fluid and crystallized intelligence: A critical experiment. Journal of Educational Psychology, 54(1), 1–22. 10.1037/h0046743 6043849

[bibr11-00332941211000667] Cattell R. (1966). The scree test for the number of factors. Multivariate Behavioral Research, 1(2), 245–276. 10.1207/s15327906mbr0102_10 26828106

[bibr12-00332941211000667] Cattell R. Horn J. (1978). A check on the theory of fluid and crystallized intelligence with description of new subtest designs. Journal of Educational Measurement, 15(3), 139–164. 10.1111/j.1745-3984.1978.tb00065.x

[bibr13-00332941211000667] Charles M. Harr B. Cech E. Hendley A. (2014). Who likes math where? Gender differences in eighth-graders’ attitudes around the world. International Studies in Sociology of Education, 24(1), 85–112. 10.1080/09620214.2014.895140

[bibr14-00332941211000667] Cheryan S. Meltzoff A. Kim S. (2011). Classrooms matter: The design of virtual classrooms influences gender disparities in computer science classes. Computers & Education, 57(2), 1825–1835. 10.1016/j.compedu.2011.02.004

[bibr15-00332941211000667] Cheryan S. Plaut V. (2010). Explaining underrepresentation: A theory of precluded interest. Sex Roles, 63(7–8), 475–488. 10.1007/s11199-010-9835-x 20930923 PMC2937137

[bibr16-00332941211000667] Cheryan S. Plaut V. Davies P. Steele C. (2009). Ambient belonging: How stereotypical cues impact gender participation in computer science. Journal of Personality and Social Psychology, 97(6), 1045–1060. 10.1037/a0016239 19968418

[bibr17-00332941211000667] Cheryan S. Ziegler S. Montoya A. Jiang L. (2017). Why are some STEM fields more gender balanced than others? Psychological Bulletin, 143(1), 1–35. 10.1037/bul0000052 27732018

[bibr18-00332941211000667] Cimpian A. Leslie S.-J. (2015). Response to comment on “expectations of brilliance underlie gender distributions across academic disciplines. Science (New York, N.Y.), 349(6246), 391. 10.1126/science.aaa9892 26206927

[bibr19-00332941211000667] Cohen L. Manion L. Morrison K. (2007). *Research methods in education* (6th ed.). Routledge.

[bibr20-00332941211000667] Courter S. Millar S. Lyons L. (1998). From the students’ point of view: Experiences in a freshman engineering design course. Journal of Engineering Education, 87(3), 283–288. 10.1002/j.2168-9830.1998.tb00355.x

[bibr21-00332941211000667] Covington K. Froyd J. (2004). Challenges of changing faculty attitudes about the underlying nature of gender inequities [Paper presentation]. *2004 American Society for Engineering Education Annual Conference & Exposition* (pp. 1–16). Texas A&M University.

[bibr22-00332941211000667] Cronin C. Roger A. (1999). Theorizing progress: Women in science, engineering, and technology in higher education. Journal of Research in Science Teaching, 36(6), 637–661. 10.1002/(SICI)1098-2736(199908)36:6<637::AID-TEA4>3.0.CO;2-9

[bibr23-00332941211000667] Dar-Nimrod I. Heine S. (2006). Exposure to scientific theories affects women’s math performance. Science (New York, N.Y.), 314(5798), 435. 10.1126/science.1131100 17053140

[bibr24-00332941211000667] Deiglmayr A. Stern E. Schubert R. (2019). Beliefs in “brilliance” and belonging uncertainty in male and female STEM students. Frontiers in Psychology, 10, 1114–1117. 10.3389/fpsyg.2019.01114 31191382 PMC6546818

[bibr25-00332941211000667] Downey G. Lucena J. (2005). National identities in multinational worlds: Engineers and “engineering cultures. International Journal of Continuing Engineering Education and Life-Long Learning, 15(3–6), 252–260. 10.1504/IJCEELL.2005.007714

[bibr26-00332941211000667] Dweck C. (1999). Self-theories: Their role in motivation, personality, and development. Psychology Press. 2130257

[bibr27-00332941211000667] Dweck C. (2006). Mindset: The new psychology of success. Random House.

[bibr28-00332941211000667] Eagly A. (2007). Female leadership advantage and disadvantage: Resolving the contradictions. Psychology of Women Quarterly, 31(1), 1–12. 10.1111/j.1471-6402.2007.00326.x

[bibr29-00332941211000667] Eagly A. Makhijani M. Klonsky B. (1992). Gender and the evaluation of leaders: A meta-analysis. Psychological Bulletin, 111(1), 3–22. 10.1037/0033-2909.111.1.3 7870858

[bibr30-00332941211000667] Eisenhart M. (2001). Educational ethnography past, present, and future: Ideas to think with. Educational Researcher, 30(8), 16–27. 10.3102/0013189X030008016

[bibr31-00332941211000667] Ellis A. Burchett W. Harrar S. Bathke A. (2017). Nonparametric inference for multivariate data: The R package NPMV. Journal of Statistical Software, 76(4), 1–18. 10.18637/jss.v076.i04

[bibr32-00332941211000667] Elmore K. Luna-Lucero M. (2017). Light bulbs or seeds? How metaphors for ideas influence judgments about genius. Social Psychological and Personality Science, 8(2), 200–208. 10.1177/1948550616667611

[bibr33-00332941211000667] Finnigan K. Corker K. (2016). Do performance avoidance goals moderate the effect of different types of stereotype threat on women’s math performance? Journal of Research in Personality, 63, 36–43. 10.1016/j.jrp.2016.05.009

[bibr34-00332941211000667] Flore P. Mulder J. Wicherts J. (2018). The influence of gender stereotype threat on mathematics test scores of Dutch high school students: A registered report. Comprehensive Results in Social Psychology, 3(2), 140–174. 10.1080/23743603.2018.1559647

[bibr35-00332941211000667] Foliano F. Rolfe H. Buzzeo J. Runge J. Wilkinson D. (2019). *Changing mindsets: Effectiveness trial*. Educational Endowment Foundation, National Institute of Economis and Social Research.

[bibr36-00332941211000667] Fromm E. McGourty J. (2001). *Measuring culture change in engineering education* [Paper presentation]. 2001 American Society for Engineering Education Annual Conference & Exposition, ASEE (pp. 1–12).

[bibr37-00332941211000667] Fry P. S. (1984). Teachers’ conceptions of students’ intelligence and intelligent functioning: A cross-sectional study of elementary, secondary and tertiary level teachers. International Journal of Psychology, 19(1–4), 457–474. 10.1080/00207598408247541

[bibr38-00332941211000667] Godfrey E. Parker L. (2010). Mapping the cultural landscape in engineering education. Journal of Engineering Education, 99(1), 5–22. 10.1002/j.2168-9830.2010.tb01038.x

[bibr39-00332941211000667] Gunderson E. Hamdan N. Sorhagen N. Esterre A. (2017). Who needs innate ability to succeed in math and literacy? Academic-domain-specific theories of intelligence about peers versus adults. Developmental Psychology, 53(6), 1188–1205. 10.1037/dev0000282 28383932

[bibr40-00332941211000667] Hallström J. Elvstrand H. Hellberg K. (2015). Gender and technology in free play in Swedish early childhood education. International Journal of Technology and Design Education, 25(2), 137–149. 10.1007/s10798-014-9274-z

[bibr41-00332941211000667] Hamamura T. (2012). Power distance predicts gender differences in math performance across societies. Social Psychological and Personality Science, 3(5), 545–548. 10.1177/1948550611429191

[bibr42-00332941211000667] Horn J. (1965). A rationale and test for the number of factors in factor analysis. Psychometrika, 30(2), 179–185. 10.1007/bf02289447 14306381

[bibr43-00332941211000667] Horn J. Cattell R. (1966). Refinement and test of the theory of fluid and crystallized general intelligences. Journal of Educational Psychology, 57(5), 253–270. 10.1037/h0023816 5918295

[bibr44-00332941211000667] Hunt J. (2016). Why do women leave science and engineering? ILR Review, 69(1), 199–226. 10.1177/0019793915594597

[bibr45-00332941211000667] Inglehart R. Welzel C. (2005). Modernization, cultural change and democracy. Cambridge University Press.

[bibr46-00332941211000667] Irish State Examinations Commission. (2019). *State examinations statistics*. https://www.examinations.ie/statistics/?l=en&mc=st&sc=r18

[bibr47-00332941211000667] Kaiser H. (1960). The application of electronic computers to factor analysis. Educational and Psychological Measurement, 20(1), 141–151. 10.1177/001316446002000116

[bibr48-00332941211000667] Kaiser H. (1974). An index of factorial simplicity. Psychometrika, 39(1), 31–36. 10.1007/BF02291575

[bibr49-00332941211000667] Kirkcaldy B. Noack P. Furnham A. Siefen G. (2007). Parental estimates of their own and their children’s intelligence. European Psychologist, 12(3), 173–180. 10.1027/1016-9040.12.3.173

[bibr50-00332941211000667] Koenig A. M. Eagly A. H. Mitchell A. A. Ristikari T. (2011). Are leader stereotypes masculine? A meta-analysis of three research paradigms. Psychological Bulletin, 137(4), 616–642. 10.1037/a0023557 21639606

[bibr51-00332941211000667] Langan A. M. Shuker D. Cullen W. R. Penney D. Preziosi R. Wheater C. P. (2008). Relationships between student characteristics and self-, peer and tutor evaluations of oral presentations. Assessment & Evaluation in Higher Education, 33(2), 179–190. 10.1080/02602930701292498

[bibr52-00332941211000667] Leahy R. Hunt T. (1983). A cognitive-developmental approach to the development of conceptions of intelligence. In Leahy R. (Ed.), The child’s construction of social inequality (pp. 135–160). Academic Press.

[bibr53-00332941211000667] Leslie S.-J. Cimpian A. Meyer M. Freeland E. (2015). Expectations of brilliance underlie gender distributions across academic disciplines. Science (New York, N.Y.), 347(6219), 262–265. 10.1126/science.1261375 25593183

[bibr54-00332941211000667] Li Y. Bates T. (2017). Does growth mindset improve children’s IQ, educational attainment or response to setbacks? 10.31235/osf.io/tsdwy

[bibr55-00332941211000667] Mann A. DiPrete T. (2016). The consequences of the national math and science performance environment for gender differences in STEM aspirations. Sociological Science, 3(1), 568–603. 10.15195/v3.a25

[bibr56-00332941211000667] Mason C. Rebok G. (1984). Psychologists’ self-perceptions of their intellectual aging. International Journal of Behavioral Development, 7(3), 255–266. 10.1177/016502548400700301

[bibr57-00332941211000667] McKenna A. Hutchinson M. Trautvetter L. (2008). *The engineer of 2020: Case studies of organizational features of effective engineering education* [Symposium]. 2008 *Research in Engineering Education Symposium*. Research in Engineering Education Network Davos, Switzerland

[bibr58-00332941211000667] Meehl P. (2006). The power of quantitative thinking. In Waller N. Yonce L. Grove W. Faust D. Lenzenweger M. (Eds.), A Paul Meehl reader: Essays on the practice of scientific psychology (pp. 433–444). Erlbaum.

[bibr59-00332941211000667] Meyer M. Cimpian A. Leslie S.-J. (2015). Women are underrepresented in fields where success is believed to require brilliance. Frontiers in Psychology, 6, 235–212. 10.3389/fpsyg.2015.00235 25814964 PMC4356003

[bibr60-00332941211000667] Miller D. Eagly A. Linn M. (2015). Women’s representation in science predicts national gender-science stereotypes: Evidence from 66 nations. Journal of Educational Psychology, 107(3), 631–644. 10.1037/edu0000005

[bibr61-00332941211000667] Mugny G. Carugati F. (1989). Social representations of intelligence. Cambridge University Press.

[bibr62-00332941211000667] Murphy T. Shehab R. Reed-Rhoads T. Foor C. Harris B. Trytten D. Walden S. Besterfield-Sacre M. Hallbeck M. S. Moor W. (2007). Achieving parity of the sexes at the undergraduate level: A study of success. Journal of Engineering Education, 96(3), 241–252. 10.1002/j.2168-9830.2007.tb00933.x

[bibr63-00332941211000667] Neisser U. (1979). The concept of intelligence. In R. Sternberg & D. Detterman (Eds.), *Human intelligence: Perspectives on its theory and measurement* (Vol. 3, Issue 3, pp. 179–189). Ablex.

[bibr64-00332941211000667] Nosek B. A. Smyth F. L. Sriram N. Lindner N. M. Devos T. Ayala A. Bar-Anan Y. Bergh R. Cai H. Gonsalkorale K. Kesebir S. Maliszewski N. Neto F. Olli E. Park J. Schnabel K. Shiomura K. Tulbure B. T. Wiers R. W. Greenwald A. G. (2009). National differences in gender-science stereotypes predict national sex differences in science and math achievement. Proceedings of the National Academy of Sciences of the United States of America, 106(26), 10593–10597. 10.1073/pnas.0809921106 19549876 PMC2705538

[bibr65-00332941211000667] O’Connor P. Goransson A. (2015). Constructing or rejecting the notion of the other in university management: The cases of Ireland and Sweden. Educational Management Administration & Leadership, 43(2), 323–340. 10.1177/1741143214523015

[bibr66-00332941211000667] Organisation for Economic Co-operation and Development. (2020). *OECD.Stat: Enrolment by field*. https://stats.oecd.org/#

[bibr67-00332941211000667] Pawley A. Schimpf C. Nelson L. (2016). Gender in engineering education research: A content analysis of research in JEE, 1998-2012. Journal of Engineering Education, 105(3), 508–528. 10.1002/jee.20128

[bibr68-00332941211000667] Pérez L. González C. Beltrán J. (2010). Parental estimates of their own and their relatives’ intelligence: A Spanish replication. Learning and Individual Differences, 20(6), 669–676. 10.1016/j.lindif.2010.09.005

[bibr69-00332941211000667] Reilly D. (2012). Gender, culture, and sex-typed cognitive abilities. PLoS One, 7(7), e39904. 10.1371/journal.pone.0039904 22808072 PMC3393715

[bibr70-00332941211000667] Rosch E. (1977). Human categorization. In Warren N. (Ed.), Studies in cross-cultural psychology (pp. 1–49). Academic Press.

[bibr71-00332941211000667] Rosch E. Mervis C. (1975). Family resemblances: Studies in the internal structure of categories. Cognitive Psychology, 7(4), 573–605. 10.1016/0010-0285(75)90024-9

[bibr72-00332941211000667] Rosch E. Mervis C. Gray W. Johnson D. Boyes-Braem P. (1976). Basic objects in natural categories. Cognitive Psychology, 8(3), 382–439. 10.1016/0010-0285(76)90013-x

[bibr73-00332941211000667] Sandström M. Stier J. Sandberg A. (2013). Working with gender pedagogics at 14 Swedish preschools. Journal of Early Childhood Research, 11(2), 123–132. 10.1177/1476718X12466205

[bibr74-00332941211000667] Sax L. (1994). Mathematical self-concept: How college reinforces the gender gap. Research in Higher Education, 35(2), 141–166. 10.2307/40196084

[bibr75-00332941211000667] Sax L. Kanny M. A. Riggers-Piehl T. Whang H. Paulson L. (2015). “But I’m not good at math”: The changing salience of mathematical self-concept in shaping women’s and men’s STEM aspirations. Research in Higher Education, 56(8), 813–842. 10.1007/s11162-015-9375-x

[bibr76-00332941211000667] Schein E. (1992). Organizational culture and leadership (2nd ed.). Jossey-Bass Publishers.

[bibr77-00332941211000667] Sendén M. G. Klysing A. Lindqvist A. Renström E. A. (2019). The (not so) changing man: Dynamic gender stereotypes in Sweden. Frontiers in Psychology, 10(37), 1–17. 10.3389/fpsyg.2019.00037 30761034 PMC6363713

[bibr78-00332941211000667] Sisk V. Burgoyne A. Sun J. Butler J. Macnamara B. (2018). To what extent and under which circumstances are growth mind-sets important to academic achievement? Two meta-analyses. Psychological Science, 29(4), 549–571. 10.1177/0956797617739704 29505339

[bibr79-00332941211000667] Skolverket. (2018). *Curriculum for the compulsory school, preschool class and school-age educare: Revised*. Swedish National Agency for Education.

[bibr80-00332941211000667] Sternberg R. (2000). The concept of intelligence. In Sternberg R. (Ed.), Handbook of intelligence (pp. 3–15). Cambridge University Press.

[bibr81-00332941211000667] Sternberg R. Conway B. Ketron J. Bernstein M. (1981). People’s conceptions of intelligence. Journal of Personality and Social Psychology, 41(1), 37–55. 10.1037/0022-3514.41.1.37

[bibr82-00332941211000667] Stevens R. O'Connor K. Garrison L. Jocuns A. Amos D. M. (2008). Becoming an engineer: Toward a three dimensional view of engineering learning. Journal of Engineering Education, 97(3), 355–368. 10.1002/j.2168-9830.2008.tb00984.x

[bibr83-00332941211000667] Stoet G. Bailey D. Moore A. Geary D. (2016). Countries with higher levels of gender equality show larger national sex differences in mathematics anxiety and relatively lower parental mathematics valuation for girls. PLoS One, 11(4), e0153857. 10.1371/journal.pone.0153857 27100631 PMC4839696

[bibr84-00332941211000667] Storage D. Horne Z. Cimpian A. Leslie S.-J. (2016). The frequency of “brilliant” and “genius” in teaching evaluations predicts the representation of women and African Americans across fields. PLoS One, 11(3), e0150194. 10.1371/journal.pone.0150194 26938242 PMC4777431

[bibr85-00332941211000667] Sultan U. Axell C. Hallström J. , (2018). Girls’ engagement in technology education: A systematic review of the literature. In Seery N. Buckley J. Canty D. Phelan J. (Eds.), PATT36 International conference: Research and practice in technology education: Perspectives on human capacity and development (pp. 231–238). Technology Education Research Group.

[bibr86-00332941211000667] Sunny C. E. Taasoobshirazi G. Clark L. Marchand G. (2017). Stereotype threat and gender differences in chemistry. Instructional Science, 45(2), 157–175. 10.1007/s11251-016-9395-8

[bibr87-00332941211000667] Swim J. Aikin K. Hall W. Hunter B. (1995). Sexism and racism: Old-fashioned and modern prejudices. Journal of Personality and Social Psychology, 68(2), 199–214. 10.1037//0022-3514.68.2.199

[bibr88-00332941211000667] Swim J. Cohen L. (1997). Overt, covert, and subtle sexism. Psychology of Women Quarterly, 21(1), 103–118. 10.1111/j.1471-6402.1997.tb00103.x

[bibr89-00332941211000667] Swim J. Mallett R. Russo-Devosa Y. Stangor C. (2005). Judgments of sexism: A comparison of the subtlety of sexism measures and sources of variability in judgments of sexism. Psychology of Women Quarterly, 29(4), 406–411. 10.1111/j.1471-6402.2005.00240.x

[bibr90-00332941211000667] Tabachnick B. Fidell L. (2007). Using multivariate statistics (5th ed.). Allyn & Bacon.

[bibr91-00332941211000667] Tiedemann J. (2000). Gender-related beliefs of teachers in elementary school mathematics. Educational Studies in Mathematics, 41(2), 191–207. 10.2307/3483189

[bibr92-00332941211000667] Tonso K. (2006). Teams that work: Campus culture, engineer identity, and social interactions. Journal of Engineering Education, 95(1), 25–37. 10.1002/j.2168-9830.2006.tb00875.x

[bibr93-00332941211000667] Torres-Guijarro S. Bengoechea M. (2017). Gender differential in self-assessment: A fact neglected in higher education peer and self-assessment techniques. Higher Education Research & Development, 36(5), 1072–1084. 10.1080/07294360.2016.1264372

[bibr94-00332941211000667] Valian V. (1998). Why so slow? The advancement of women. MIT Press.

[bibr95-00332941211000667] Velicer W. Eaton C. Fava J. (2000). Construct explication through factor or component analysis: A review and evaluation of alternative procedures for determining the number of factors of components. In Goffin R. ?0026; Helmes E. (Eds.), Problems and solutions in human assessment: Honoring Douglas N. Jackson at seventy (pp. 47–71). Kluwer Academic.

[bibr96-00332941211000667] Verdín D. Godwin A. Kirn A. Benson L. Potvin G. (2018). Engineering women’s attitudes and goals in choosing disciplines with above and below average female representation. Social Sciences, 7(3), 44–25. 10.3390/socsci7030044

[bibr97-00332941211000667] Volkwein J. F. Lattuca L. Terenzini P. Strauss L. Sukhbaatar J. (2004). Engineering change: A study of the impact of EC2000*. International Journal of Engineering Education, 20(3), 318–328. https://www.ijee.ie/articles/Vol20-3/IJEE2497.pdf

[bibr98-00332941211000667] Wang M. Degol J. (2017). Gender gap in science, technology, engineering, and mathematics (STEM): Current knowledge, implications for practice, policy, and future directions. Educational Psychology Review, 29(1), 119–140. 10.1007/s10648-015-9355-x 28458499 PMC5404748

[bibr99-00332941211000667] Wiebe E. Unfried A. Faber M. (2018). The relationship of STEM attitudes and career interest. Eurasia Journal of Mathematics, Science and Technology Education, 14(10), 1–17. 10.29333/ejmste/92286

[bibr100-00332941211000667] Wigfield A. Eccles J. (2000). Expectancy-value theory of achievement motivation. Contemporary Educational Psychology, 25(1), 68–81. 10.1006/ceps.1999.1015 10620382

[bibr101-00332941211000667] Yoder B. (2017). *Engineering by the numbers*. https://www.asee.org/documents/papers-and-publications/publications/college-profiles/2017-Engineering-by-Numbers-Engineering-Statistics.pdf

[bibr102-00332941211000667] Yussen S. Kane P. (1983). Children’s ideas about intellectual ability. In Leahy R. (Ed.), The child’s construction of social inequality (pp. 109–134). Academic Press.

