# Supplementary material for: Exploring the Prototypical Definitions of Intelligent Engineers Held by Irish and Swedish Higher Education Engineering Students
Source: Psychol Rep. 2021 Mar 12;125(3):1397–437. doi: 10.1177/00332941211000667 (PMC9136481; doi:10.1177/00332941211000667)
Supplement: sj-pdf-1-prx-10.1177_00332941211000667 - Supplemental material for Exploring the Prototypical Definitions of Intelligent Engineers Held by Irish and Swedish Higher Education Engineering Students [file sj-pdf-1-prx-10.1177_00332941211000667.pdf]

Independent EFA solutions for the Irish and Swedish data

In comparing the factor structures between the EFA analysis where the responses from both the Irish and Swedish participants were included and the pursuant EFA analyses where the data from the Irish and Swedish participants were included separately, the EFA solution where the data from both countries were included will henceforth be referred to as the 'consolidated factor structure'. To examine the EFA structures using both the Irish and Swedish data separately, identical processes of determining the factorability of the data, the number of factors to extract and conducting an EFA to those used in determining the consolidated factor structure were implemented. Additionally, as with the previous EFA, factors were theoretically interpreted based only on characteristics which loaded on them that had pattern coefficients greater than .4 and less than -.4.

For the Irish sample, of the 3916 correlations in the correlation matrix, 314 were greater than or equal to .3. An examination of the anti-image correlation matrix revealed that 73 of the 89 anti-image correlations were above .5, and the off-diagonal elements were mostly small (M = -.0104, SD = .1388). The KMO measure of sampling adequacy was .617, above the recommended value of .6 (Kaiser, 1974), and Bartlett's test of sphericity was significant (χ^2 (3916) = 7356.199, $p < .001$). Therefore, there was a reasonable level of factorability within the data. A parallel analysis (Horn, 1965) suggested a seven factor solution for the Irish sample Figure S1. The EFA solution is presented in Table S3.

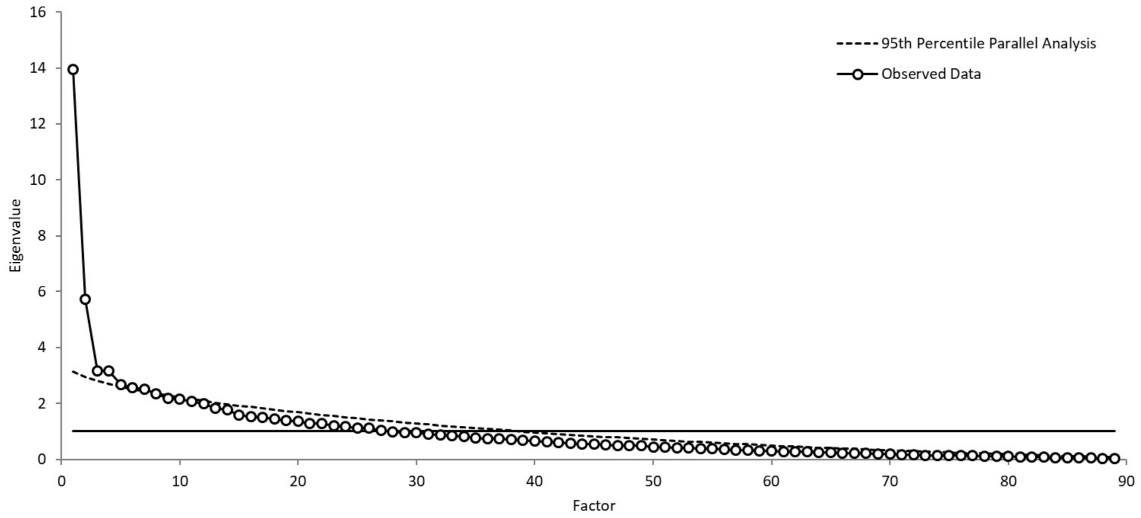

FIGURE S1 Factor eigenvalues and parallel analysis for the EFA with data from the Irish sample only.

TABLE S3 Seven factor oblique EFA solution inclusive of data from Irish participants only.

Characteristic	F1	F2	F3	F4	F5	F6	F7	*h*[2]
Able to understand complex information	**.733** (.724)	.093 (.037)	-.095 (.161)	-.034 (.189)	-.002 (.213)	.201 (.279)	.032 (.220)	.793
Solution orientated	**.531** (.547)	-.012 (-.045)	-.033 (.146)	-.034 (.112)	.058 (.181)	.086 (.153)	.026 (.166)	.750
Methodical	**.529** (.573)	.015 (.048)	-.019 (.205)	.159 (.305)	.073 (.245)	.116 (.237)	-.058 (.145)	.753
Practically orientated	**.510** (.505)	.202 (.175)	.036 (.202)	.020 (.213)	.115 (.279)	-.038 (.113)	-.066 (.138)	.692
Responsible	**.490** (.571)	.069 (.088)	-.118 (.069)	.212 (.380)	-.167 (.079)	-.048 (.007)	**.440** (.576)	.782
Realistic	**.478** (.526)	.094 (.108)	.015 (.192)	.022 (.211)	.209 (.340)	-.112 (.037)	.070 (.269)	.776
Spatial ability	**.459** (.515)	.055 (.034)	.205 (.374)	-.098 (.117)	.072 (.262)	.168 (.297)	-.031 (.125)	.754
Competent in technology	**.452** (.537)	-.067 (-.054)	.265 (.429)	.023 (.176)	.118 (.272)	.102 (.262)	-.137 (.05)	.758
Problem solving	**.437** (.522)	-.208 (-.244)	.102 (.247)	-.231 (-.081)	.110 (.184)	.123 (.154)	.173 (.239)	.760
Quick thinking	**.434** (.473)	.111 (.065)	.092 (.227)	-.059 (.140)	-.081 (.127)	.080 (.154)	.175 (.287)	.749
Visionary	**.418** (.461)	.054 (.071)	.259 (.376)	.344 (.410)	-.072 (.121)	-.042 (.151)	-.278 (-.045)	.753
Disorganised	.189 (.025)	**.756** (.616)	.031 (.032)	-.240 (.026)	-.027 (.162)	-.082 (.030)	-.080 (.020)	.717
Lazy	.062 (-.123)	**.710** (.607)	-.088 (-.119)	-.033 (.115)	-.105 (.035)	-.114 (-.039)	-.150 (-.073)	.838
Stressed	.108 (-.002)	**.691** (.596)	.094 (.074)	-.041 (.176)	-.164 (.066)	-.126 (-.008)	.004 (.101)	.838
Easily bored	.108 (.008)	**.543** (.469)	.129 (.127)	-.074 (.104)	-.023 (.136)	-.049 (.078)	-.178 (-.071)	.781
Stubborn	.112 (.037)	**.538** (.490)	-.014 (.023)	-.071 (.137)	-.036 (.145)	-.036 (.051)	.058 (.141)	.727
Quiet	-.043 (-.054)	**.521** (.606)	-.087 (.035)	.053 (.285)	.063 (.281)	.191 (.288)	.072 (.164)	.720
Lacking social skills	-.040 (-.125)	**.520** (.498)	.017 (-.018)	.127 (.223)	-.086 (.047)	-.173 (-.072)	-.072 (.011)	.742
Strange	-.274 (-.246)	**.484** (.570)	.144 (.183)	-.084 (.145)	.076 (.270)	.221 (.323)	.060 (.088)	.783
Creative	.232 (.327)	.033 (.051)	**.494** (.543)	.152 (.256)	.018 (.190)	-.062 (.156)	-.233 (-.046)	.769
Desire to learn	.027 (.170)	.113 (.159)	**.479** (.471)	.327 (.420)	-.329 (-.043)	-.079 (.076)	.149 (.252)	.760
Creatively brave	.021 (.110)	.180 (.202)	**.469** (.465)	.023 (.170)	.042 (.206)	-.099 (.093)	-.065 (.056)	.696
Diligent	.105 (.320)	-.046 (.077)	**.435** (.550)	.168 (.342)	.020 (.262)	.062 (.245)	.175 (.314)	.777
Decision making skills	.046 (.189)	-.137 (-.108)	**.417** (.401)	-.034 (.035)	.145 (.185)	-.226 (-.087)	.087 (.164)	.681
Supportive	-.078 (.130)	-.119 (.210)	-.015 (.198)	**.601** (.623)	.362 (.450)	.007 (.204)	-.043 (.169)	.799
Positive	-.006 (.128)	.033 (.231)	.058 (.169)	**.597** (.603)	.006 (.164)	-.108 (.052)	.019 (.192)	.707
Motivated	-.009 (.171)	-.203 (-.010)	.178 (.278)	**.486** (.457)	.058 (.156)	-.040 (.100)	-.011 (.128)	.715
Healthy	-.036 (.113)	.154 (.386)	.118 (.300)	**.443** (.575)	.130 (.356)	.156 (.351)	-.039 (.150)	.847
Ambitious	.105 (.242)	.099 (.227)	.111 (.220)	**.416** (.515)	-.108 (.124)	-.092 (.044)	.241 (.385)	.709
Detail orientated	.276 (.329)	-.031 (.052)	-.015 (.175)	-.148 (.011)	**.577** (.550)	.022 (.169)	-.157 (.013)	.824
Thoughtful	.328 (.385)	.147 (.251)	-.139 (.060)	.096 (.276)	**.440** (.505)	-.212 (-.038)	.083 (.302)	.765
Economic	.053 (.199)	-.079 (.072)	.029 (.169)	.011 (.141)	**.437** (.452)	-.089 (.038)	.168 (.284)	.711
Empathetic	.089 (.126)	.276 (.377)	.044 (.154)	.075 (.240)	**.409** (.475)	-.189 (.015)	-.093 (.099)	.678
Funny	-.316 (-.140)	.186 (.442)	.231 (.343)	.174 (.347)	**.404** (.531)	.073 (.286)	.003 (.135)	.777

Characteristic	F1	F2	F3	F4	F5	F6	F7	h^2
Competent in mathematics	.055 (.166)	-.390 (-.286)	-.031 (.138)	.054 (.046)	-.096 (-.024)	**.663** (.574)	-.005 (-.051)	.749
Competence in mechanics	.281 (.364)	-.011 (.023)	.006 (.234)	-.217 (.026)	.002 (.212)	**.585** (.577)	.190 (.218)	.749
Competence in physics	.224 (.258)	-.037 (.021)	-.184 (.044)	-.148 (.014)	.100 (.212)	**.555** (.518)	.064 (.087)	.797
Can make complex systems	**.430** (.442)	.079 (.085)	.044 (.257)	.130 (.278)	-.186 (.071)	**.483** (.542)	-.162 (-.030)	.824
Competence in science	.368 (.432)	.170 (.191)	.057 (.278)	-.076 (.198)	-.057 (.228)	**.408** (.476)	.165 (.272)	.757
Dedicated	-.029 (.235)	-.111 (.053)	.332 (.417)	.322 (.443)	-.147 (.110)	-.024 (.095)	**.491** (.567)	.843
Disciplined	.072 (.235)	.122 (.185)	.269 (.347)	-.122 (.131)	.137 (.323)	-.100 (.033)	**.440** (.519)	.781
α	.835	.802	.632	.682	.613	.741	.589	
Eigenvalue	13.947	5.721	3.156	3.149	2.667	2.571	2.503	
% of Variance	15.671	6.428	3.546	3.538	2.997	2.889	2.812	
Factor correlations								
F1	–							
F2	-.109	–						
F3	.264	.067	–					
F4	.218	.350	.231	–				
F5	.216	.324	.296	.262	–			
F6	.131	.188	.299	.227	.275	–		
F7	.263	.158	.154	.271	.250	.023	–	

Note. Factor pattern coefficients (structure coefficients) based on maximum likelihood extraction with promax rotation (k = 4). Salient pattern coefficients presented in bold (pattern coefficient > .4 and > -.4) only were used to calculate Cronbach's α. h^2 = communality.

The first factor which was extracted is loaded most heavily on by the characteristic of being able to understand complex information. This is then followed by five qualitatively similar personality characteristics; being solution orientation, methodical, practical orientated, responsible and realistic. While not exactly the same, it shares many of the higher loading characteristics as Factor 5 'Reasoning' in the consolidated factor structure. The second factor which was extracted very closely resembles Factor 6 'Negative attributes' from the consolidated factor structure as it is most highly loaded on by the characteristics of being disorganised, lazy and stressed. The third factor which was extracted is most highly loaded on by characteristics associated with being creative and having a desire to learn, which taken together do not clearly reflect any of the factors from the consolidated factor structure. The fourth factor is primarily associated with being supportive, positive and being driven. Again, it does not clearly resemble one of the earlier factors but can be seen to have some alignment Factor 2 'Conscientiousness' and Factor 3 'Drive'. The fifth factor, however, more clearly reflects Factor 2 'Conscientiousness' from the consolidated factor structure as it is most highly loaded on by the characteristics of detail orientated, thoughtful, economic and empathetic and

as the sixth extracted factor is loaded on by the characteristics relating to competence in discipline areas, it closely resembles the previous Factor 4 ‘Discipline Knowledge’. The final factor to be extracted is only loaded on highly by the three characteristics of being responsible, dedicated and disciplined. It does have low reliability ($\alpha = .589$) but appears to be reflective of Factor 3 ‘Drive’, at least more so than the fourth extracted factor. Based on these results, while there was not exact alignment with the consolidated factor structure, there were many similarities particularly with the factors associated with negative attributes, conscientiousness, discipline knowledge, and to a lesser degree with the factors of practical problem solving, and drive. Notably, Factor 5 ‘Reasoning’ which had a significant main effect of the participants’ country and was rated more important by the Swedish sample was not clearly observed in the EFA solution from the Irish sample and neither was Factor 7 ‘Inquisitiveness’.

For the Swedish sample, of the 3916 correlations in the correlation matrix, 395 were greater than or equal to .3. An examination of the anti-image correlation matrix revealed that 86 of the 89 anti-image correlations were above .5, and the off-diagonal elements were mostly small (M = -.0105, SD = .1193). The KMO measure of sampling adequacy was .714, above the recommended value of .6 (Kaiser, 1974), and Bartlett’s test of sphericity was significant (χ^2 (3916) = 8084.649, $p < .001$). Therefore, there was a reasonable level of factorability within the data. A parallel analysis suggested a five factor solution for the Swedish sample (Figure S2). Please see Table S4 for the full EFA solution.

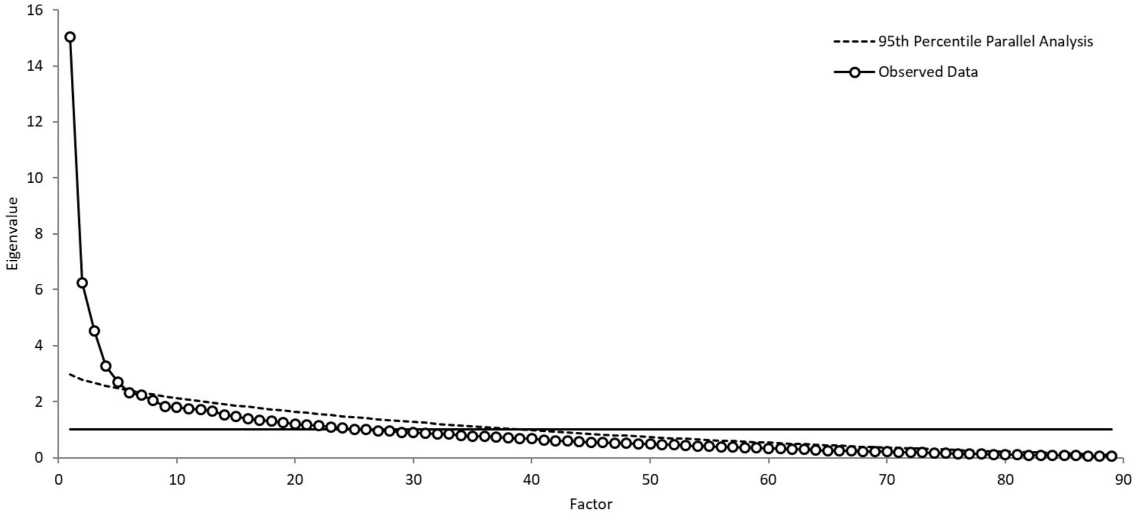

FIGURE S2 Factor eigenvalues and parallel analysis for the EFA with data from the Swedish sample only.

TABLE S4 Seven factor oblique EFA solution inclusive of data from Swedish participants only.

Characteristic	F1	F2	F3	F4	F5	h^2
Ethical	**.798** (.624)	-.012 (.015)	.093 (.054)	-.372 (-.049)	-.096 (.077)	.785
Empathetic	**.697** (.647)	-.221 (-.125)	-.002 (.041)	-.047 (.194)	.007 (.161)	.753
Humble	**.671** (.613)	-.072 (-.022)	.161 (.205)	.055 (.281)	-.261 (.008)	.811
Supportive	**.616** (.697)	.002 (.120)	.116 (.273)	.295 (.533)	-.158 (.190)	.805
Open minded	**.613** (.597)	-.032 (.052)	.040 (.111)	-.009 (.236)	-.036 (.177)	.644
Honest	**.595** (.554)	.083 (.125)	.140 (.196)	.006 (.241)	-.213 (.061)	.723
Reliable	**.584** (.644)	.089 (.193)	-.087 (.063)	.119 (.355)	.029 (.290)	.761
Self-control	**.566** (.634)	-.067 (.062)	-.001 (.132)	.156 (.387)	.040 (.272)	.791
Good communicator	**.546** (.623)	-.094 (.035)	-.043 (.098)	.197 (.402)	.047 (.267)	.736
Nice	**.539** (.596)	-.192 (-.060)	.164 (.262)	.198 (.418)	-.055 (.175)	.827
Thoughtful	**.539** (.552)	.200 (.247)	-.038 (.078)	.076 (.283)	-.116 (.163)	.699
Responsible	**.520** (.588)	.109 (.199)	-.112 (.042)	.164 (.360)	.004 (.259)	.738
Reflective	**.496** (.524)	.236 (.312)	-.266 (-.140)	-.110 (.121)	.215 (.377)	.775
Mature	**.477** (.541)	.115 (.245)	.101 (.208)	-.040 (.261)	.136 (.355)	.730
Good collaborator	**.475** (.591)	-.200 (-.039)	-.099 (.055)	.238 (.417)	.175 (.335)	.727
Good social skills	**.475** (.597)	-.060 (.120)	.061 (.205)	.110 (.388)	.218 (.417)	.742
Reasonable	**.472** (.488)	.332 (.376)	-.140 (-.023)	-.067 (.157)	.041 (.270)	.743
Competence in physics	-.018 (.051)	**.673** (.623)	.219 (.318)	.138 (.229)	-.335 (-.010)	.780
Competence in science	.006 (.111)	**.654** (.625)	.140 (.280)	.228 (.311)	-.294 (.047)	.793
Able to understand complex information	-.037 (.081)	**.650** (.650)	-.088 (.058)	.059 (.154)	.030 (.244)	.700
Competence in mathematics	-.166 (-.067)	**.632** (.609)	.156 (.247)	.059 (.134)	-.115 (.102)	.736
Competence in technology	.090 (.185)	**.629** (.645)	.072 (.202)	.016 (.191)	-.042 (.232)	.742
Competence in mechanics	-.096 (.052)	**.625** (.640)	.134 (.278)	.162 (.268)	-.085 (.185)	.791
Educated	.077 (.143)	**.562** (.559)	.109 (.208)	.010 (.157)	-.105 (.147)	.696
Intelligent	-.262 (-.087)	**.523** (.578)	.032 (.155)	.018 (.112)	.242 (.345)	.753
Can make complex systems	-.249 (-.047)	**.484** (.587)	.174 (.296)	-.014 (.151)	.311 (.426)	.733
Logical	-.097 (.028)	**.439** (.467)	-.138 (-.013)	.050 (.108)	.167 (.272)	.684
Interested in engineering	.089 (.197)	**.424** (.478)	.136 (.252)	.054 (.228)	.012 (.241)	.601
Field specific knowledge	.037 (.091)	**.422** (.435)	-.145 (-.062)	-.117 (-.009)	.170 (.257)	.676
Methodical	.148 (.273)	**.405** (.457)	-.167 (.000)	.125 (.245)	.105 (.305)	.667
Strange	.078 (.175)	-.035 (.105)	**.636** (.649)	.018 (.270)	.009 (.173)	.663
Nerdy	-.044 (.036)	.059 (.150)	**.586** (.583)	.007 (.192)	-.045 (.094)	.724
Easily bored	.008 (.068)	.026 (.126)	**.556** (.539)	-.083 (.131)	.027 (.135)	.728
Stressed	.009 (.086)	.030 (.126)	**.553** (.557)	.019 (.209)	-.044 (.101)	.696
Pessimistic	.070 (.134)	.129 (.19)	**.542** (.566)	.103 (.276)	-.211 (.016)	.593

Characteristic	F1	F2	F3	F4	F5	h^2
Craft skill	.000 (.170)	.042 (.249)	**.528** (.581)	-.105 (.215)	.364 (.461)	.691
Lazy	-.043 (-.040)	.030 (.092)	**.526** (.456)	-.220 (-.034)	.036 (.073)	.681
Lacking social skills	-.015 (.086)	.074 (.192)	**.493** (.513)	-.040 (.176)	.098 (.216)	.720
Has a variety of areas of interest	.123 (.228)	-.032 (.151)	**.428** (.453)	-.179 (.133)	.343 (.409)	.719
Funny	.147 (.370)	-.092 (.113)	**.424** (.571)	.353 (.574)	.088 (.325)	.778
General knowledge	.213 (.249)	-.024 (.113)	**.417** (.407)	-.236 (.066)	.212 (.292)	.675
Ambitious	-.042 (.225)	.007 (.131)	.010 (.235)	**.659** (.643)	-.012 (.205)	.680
Good work ethic	-.010 (.272)	.087 (.228)	-.083 (.166)	**.597** (.618)	.108 (.321)	.795
Motivated	.112 (.319)	.056 (.154)	-.146 (.070)	**.534** (.541)	.005 (.216)	.695
Determined	.004 (.228)	.174 (.261)	-.099 (.118)	**.520** (.522)	.002 (.221)	.673
Positive	.297 (.505)	-.134 (.045)	.076 (.274)	**.465** (.612)	.077 (.313)	.807
Quick thinking	-.284 (-.049)	.219 (.381)	.159 (.274)	.013 (.166)	**.490** (.505)	.761
Intuitive	-.118 (.087)	.094 (.254)	.021 (.146)	.055 (.194)	**.465** (.480)	.642
Adaptable	.080 (.210)	-.165 (-.007)	-.037 (.045)	.018 (.152)	**.429** (.397)	.640
Creatively brave	-.111 (.118)	-.148 (.045)	.127 (.250)	.217 (.325)	**.404** (.416)	.643
Leadership skills	.074 (.335)	.017 (.229)	.024 (.225)	.273 (.455)	**.403** (.536)	.768
α	.901	.865	.821	.725	.610	
Eigenvalue	15.042	6.253	4.513	3.274	2.694	
% of Variance	16.902	7.026	5.071	3.379	3.027	
Factor correlations						
F1	–					
F2	.148	–				
F3	.144	.191	–			
F4	.408	.202	.352	–		
F5	.356	.349	.224	.345	–	

Factor pattern coefficients (structure coefficients) based on maximum likelihood extraction with promax rotation (k = 4). Salient pattern coefficients presented in bold (pattern coefficient > .4 and > -.4) only were used to calculate Cronbach's α. h^2 = communality.

The EFA solution from the Swedish sample, while a five factor solution as opposed to a seven factor solution, has factors with a clearer alignment with the consolidated factor structure than the Irish sample EFA solution. All factors also have acceptable reliability (α > .6). The first factor which was extracted is most heavily loaded on by the characteristics of being ethical, empathetic, humble, supportive, open-minded and honest, and appears to align with Factor 2 'Conscientiousness' from the consolidated factor structure. The second factor from the Swedish sample is loaded on most heavily by the characteristics relating to disciplinary competence, resembling Factor 4 'Discipline knowledge, from the consolidated factor structure. The third extracted factor is primarily described by the characteristics of being strange, nerdy, easily bored, stressed and pessimistic, reflecting Factor 6 'Negative attributes' and the fourth extracted factor pertains to the characteristics of being ambitious, having a good work ethic, and being motivated, determined and positive, and is therefore very similar to F3 'Drive' from the consolidated factor structure. The final factor which was extracted relates to

the five characteristics of being quick thinking, intuitive, adaptable, creatively brave and having leadership skills. All of these except for being adaptable were characteristics which loaded on F1 ‘Practical problem solving’ in the consolidated factor structure. Being adaptable did not load heavily on any of the factors in that solution but being resourceful, which is arguably similar, also loaded on F1 ‘Practical problem solving’, and therefore these factors appear to be describing a similar construct.
