# Supplementary material for: Exploring the Prototypical Definitions of Intelligent Engineers Held by Irish and Swedish Higher Education Engineering Students
Source: Psychol Rep. 2021 Mar 12;125(3):1397–437. doi: 10.1177/00332941211000667 (PMC9136481; doi:10.1177/00332941211000667)
Supplement: sj-pdf-2-prx-10.1177_00332941211000667 - Supplemental material for Exploring the Prototypical Definitions of Intelligent Engineers Held by Irish and Swedish Higher Education Engineering Students [file sj-pdf-2-prx-10.1177_00332941211000667.pdf]

TABLE S1 Codebook with example statements.

Code	Example statements
Ability to apply knowledge	Knowledge of how to apply what you have learned; Ability to apply theoretical knowledge to practical problems.
Ability to find relevant information	The ability to find information; Know where to find sought-after information.
Able to multitask	Good at multitasking.
Able to think abstractly	Abstraction; Good ability to think abstract; Able to abstract problems so that they become more gripable.
Able to understand complex information	Ability to understand complicated relationships; Understand so well that it can account for an understandable explanation; Able to decode information.
Adaptable	Adaptability; Adaptive; Can adapt; Flexible.
*Aggressive	Aggressive.
Ambitious	Ambitious.
Analytical	Analytical; Analytical ability; Break down complex systems into smaller components that can be more easily analysed.
Can make complex systems	Be able to create complicated systems.
Cautious	Prevention; Consider before doing anything.
Charismatic	Charisma; Charismatic.
Competent in mathematics	Good knowledge in mathematics; Good at mathematics.
*Competent in mechanics	Mechanics; Understanding of mechanics.
Competent in physics	Physicist; Basic knowledge in physics.
Competent in science	Mediation of science; Scientific.
Competent in technology	Good at technology; Technically talented.
Competitive	Competitive; Fighting spirit.
Craft skill	Good "craft" ability; Good practical skills.
Creative	Creativity; Creative thinking; easy to get many quick ideas others would call creative.
Creatively brave	Fearless to test solutions; Dare to stretch the boundaries.
Critical thinking	Source critical; Critical; Critical thinking.
Curious	Curious; Curiosity.
Decision making skills	Ability to make difficult decisions; Actionable; Good estimates.
Dedicated	Dedication; Dedicated; Perseverance.
Desire to learn	Constantly learn more/develop; The quest to always continue to learn/develop; Willing to learn; Develop their thinking for the better all the time.
Detail orientated	Meticulous; Accuracy; Eye for detail; Feeling for detail.
Determined	Determined; Determination.
Diligent	Documents the work carefully; Diligent.
Disciplined	Discipline; Disciplined; Focussed.
*Disorganised	Disorganised.
*Easily bored	Easy gets bored.
Economic	Economic.
Educated	Educated.
Efficient	Efficiency; Effective/efficient.
Empathetic	Ability to understand the needs of others; Can "get acquainted with the shoes of others" for understanding several perspectives.
Ethical	Can put their work in an ethical perspective; Morality.
Field specific knowledge	In-depth knowledge in the field; Knowledgeable in their field of work; Expertise in the subject area.
Foresight	Long-term-thinking; See problems before it arises
*Funny	Funny; Have a sense of humour; Witty.
General knowledge	Generally formed/generally knowledgeable; Broad knowledge.
Good at learning	Ability to effectively absorb new knowledge; Ability to familiarize themselves with new systems; Knowledge acquisition; Fast learner.
Good collaborator	Good at working in groups and projects; Collaborative.
Good communicator	Ability to communicate technology in an understandable way; Good communication; Communication skills.
Good social skills	Social skills; Socially competent; EQ; Social ability.
Good work ethic	Good work ethics; Productive.
Has a variety of areas of interest	Has one or more hobby, Likes to contribute in many areas.
Have a large contact network	Large contact network.
Healthy	Healthy.

Code	Example statements
*Honest	Honest.
Humble	Humble; Prestigeless.
Independent	Own thinking/Able to have own ideas; Independence; Self-propelled.
Intelligent	Whiz-kid; IQ; High IQ; Clever; Smart.
Interested in engineering	Deep interest in their area; Interested in their profession/area; Technically interested.
Intuitive	Intuitive; Strong intuition for the relevant subject.
*Lacking social skills	Lack of social skills.
Lazy	Lazy; Do not always study.
Leadership skills	Leadership.
Logical	Good on logical thinking; Logic; Rational.
Mature	Mature.
Methodical	Methodical; Structured; Systematic.
Motivated	Motivation; Motivated; Enthusiastic; Passionate; Driving.
Nerdy	Nerdy.
Nice	Nice; Friendly.
Open minded	Openness; Open to criticism; Open minded.
Organised	Determine how to plan; Ability to plan own work; Well prepared; Organized.
Pessimistic	Someone who is a bit pessimistic (good for prevention of errors).
Positive	Belief in the future; Positive; Positive towards challenges.
Practically orientated	Thing orientated; Inventive; Practical.
*Pragmatic	Hands on; Pragmatic.
Problem solving	Good problem solving ability; Good problem solver; Effective problem solving.
Quick thinking	Fast thinking; Quick; Quick ideas; Quick understanding.
Quiet	Quiet.
Realistic	Adds perspective in discussions; Realistic.
Reasonable	Reasonable; Can settle disputes.
Reflective	Reflective.
Reliable	Reliable; Time-conscious; Consistent.
Resourceful	Resourcefulness; Improvisation ability.
Responsible	Responsible; Takes on great responsibility.
Self-aware	Knowing what it is you cannot do and then able ask someone who knows about help; Don't take on things that they cannot handle.
Self-control	Stress management; Patience; Stable; Maintains concentration even when not understood.
Solution orientated	Target focussed; Solution orientated; Impact thinking; Solution focussed.
Spatial ability	Spatial intelligence; Spatial understanding; Three and multidimensional thinking and visualizing.
*Strange	Strange.
Stressed	Stressed.
Stubborn	Stubborn.
Supportive	Helps and lifts (encourages) other workers; Supporting; Appropriate guidance; Unselfish.
Thoughtful	Thoughtful; Deep thinking.
Visionary	Vision; Can see an overall picture; Have future vision and see opportunities.

Note: * = Code created when coding the data from the Irish sample.
