# Supplementary material for: Exploring the Prototypical Definitions of Intelligent Engineers Held by Irish and Swedish Higher Education Engineering Students
Source: Psychol Rep. 2021 Mar 12;125(3):1397–437. doi: 10.1177/00332941211000667 (PMC9136481; doi:10.1177/00332941211000667)
Supplement: sj-pdf-3-prx-10.1177_00332941211000667 - Supplemental material for Exploring the Prototypical Definitions of Intelligent Engineers Held by Irish and Swedish Higher Education Engineering Students [file sj-pdf-3-prx-10.1177_00332941211000667.pdf]

TABLE S2 Results of Shapiro-Wilk tests of univariate normality prior to multivariate testing of Survey 2 data.

Test No.	IV: Country	IV: Gender	DV: Factor	*W*	*p*
1	Ireland	Female	F1	0.97003	0.6026
2	Sweden	Female	F1	0.97888	0.3720
3	Ireland	Male	F1	0.991479	0.6661
4	Sweden	Male	F1	0.986694	0.3556
5	Ireland	Female	F2	0.972631	0.6724
6	Sweden	Female	F2	0.959832	0.0433
7	Ireland	Male	F2	0.989657	0.4980
8	Sweden	Male	F2	0.986492	0.3431
9	Ireland	Female	F3	0.949741	0.2113
10	Sweden	Female	F3	0.967553	0.1051
11	Ireland	Male	F3	0.947007	0.0001
12	Sweden	Male	F3	0.980384	0.1083
13	Ireland	Female	F4	0.949054	0.2033
14	Sweden	Female	F4	0.941754	0.0059
15	Ireland	Male	F4	0.976193	0.0305
16	Sweden	Male	F4	0.950023	0.0005
17	Ireland	Female	F5	0.930818	0.0724
18	Sweden	Female	F5	0.966351	0.0915
19	Ireland	Male	F5	0.955353	0.0005
20	Sweden	Male	F5	0.973418	0.0279
21	Ireland	Female	F6	0.949687	0.2107
22	Sweden	Female	F6	0.933193	0.0025
23	Ireland	Male	F6	0.973302	0.0166
24	Sweden	Male	F6	0.903641	0.0000
25	Ireland	Female	F7	0.865432	0.0024
26	Sweden	Female	F7	0.931166	0.0020
27	Ireland	Male	F7	0.929693	0.0000
28	Sweden	Male	F7	0.944853	0.0002

Note: IV = Independent variable. DV = Dependent variable.
